# Necrotic Cells Actively Attract Phagocytes through the Collaborative Action of Two Distinct PS-Exposure Mechanisms

**DOI:** 10.1371/journal.pgen.1005285

**Published:** 2015-06-10

**Authors:** Zao Li, Victor Venegas, Yuji Nagaoka, Eri Morino, Prashant Raghavan, Anjon Audhya, Yoshinobu Nakanishi, Zheng Zhou

**Affiliations:** 1 Verna and Marrs McLean Department of Biochemistry and Molecular Biology, Baylor College of Medicine, Houston, Texas, United States of America; 2 the Graduate School of Natural Science and Technology, Kanazawa University, Shizenken, Kakuma-machi, Kanazawa, Ishikawa, Japan; 3 the School of Pharmacy, Kanazawa University, Shizenken, Kakuma-machi, Kanazawa, Ishikawa, Japan; 4 Department of Biomolecular Chemistry, University of Wisconsin-Madison, School of Medicine and Public Health, Madison, Wisconsin, United States of America; 5 the Graduate School of Medical Sciences, Kanazawa University, Shizenken, Kakuma-machi, Kanazawa, Ishikawa, Japan; The University of Texas Health Science Center at Houston, UNITED STATES

## Abstract

Necrosis, a kind of cell death closely associated with pathogenesis and genetic programs, is distinct from apoptosis in both morphology and mechanism. Like apoptotic cells, necrotic cells are swiftly removed from animal bodies to prevent harmful inflammatory and autoimmune responses. In the nematode *Caenorhabditis elegans*, gain-of-function mutations in certain ion channel subunits result in the excitotoxic necrosis of six touch neurons and their subsequent engulfment and degradation inside engulfing cells. How necrotic cells are recognized by engulfing cells is unclear. Phosphatidylserine (PS) is an important apoptotic-cell surface signal that attracts engulfing cells. Here we observed PS exposure on the surface of necrotic touch neurons. In addition, the phagocytic receptor CED-1 clusters around necrotic cells and promotes their engulfment. The extracellular domain of CED-1 associates with PS *in vitro*. We further identified a necrotic cell-specific function of CED-7, a member of the ATP-binding cassette (ABC) transporter family, in promoting PS exposure. In addition to CED-7, anoctamin homolog-1 (ANOH-1), the *C*. *elegans* homolog of the mammalian Ca^2+^-dependent phospholipid scramblase TMEM16F, plays an independent role in promoting PS exposure on necrotic cells. The combined activities from CED-7 and ANOH-1 ensure efficient exposure of PS on necrotic cells to attract their phagocytes. In addition, CED-8, the *C*. *elegans* homolog of mammalian Xk-related protein 8 also makes a contribution to necrotic cell-removal at the first larval stage. Our work indicates that cells killed by different mechanisms (necrosis or apoptosis) expose a common “eat me” signal to attract their phagocytic receptor(s); furthermore, unlike what was previously believed, necrotic cells actively present PS on their outer surfaces through at least two distinct molecular mechanisms rather than leaking out PS passively.

## Author Summary

Necrosis is a type of cell death often caused by cell injury and is linked to human diseases including neuron degeneration, stroke, and cancer. Necrotic cells undergo distinct morphological changes, including swelling, before being engulfed and degraded by engulfing cells. The clearance of necrotic cells from animal bodies is important for wound healing and for preventing harmful inflammatory and autoimmune responses. However, the mechanisms by which necrotic cells are removed remain elusive. We study the recognition of necrotic neurons in the nematode *C*. *elegans*. There is a common belief that the plasma membrane of necrotic cells are ruptured, allowing the detection of phosphatidylserine (PS), a so-called “eat me” signal molecule, by specific transmembrane receptors on the surface of engulfing cells. Contrary to this belief, we found that necrotic neurons actively present PS to their outer surface through two parallel molecular mechanisms, one of which is shared by cells undergoing apoptosis, a “cell suicide” event, whereas the other is unique to necrotic cells. Ca^2+^-influx, a key factor that triggers necrosis, is implicated in activating a unique PS-scramblase. Our findings reveal novel necrotic cell-specific “eat me” signal-exposure mechanisms and indicate that cells that die through different mechanisms (necrosis and apoptosis) utilize both common and unique mechanisms to attract engulfing cells. They further demonstrate that *C*. *elegans* is an effective model system for studying the fate of necrotic cells.

## Introduction

Cell death during animal development and under pathological conditions is important for removing unwanted cells that are often harmful. Necrosis and apoptosis are two morphologically distinct types of cell death events. Whereas cells undergoing apoptosis display features such as cytoplasm shrinkage, chromatin condensation, nuclear DNA fragmentation, and well-maintained plasma membrane integrity, necrotic cells display cell and organelle swelling, excessive intracellular membranes, and the eventual rupture of intracellular and plasma membranes (reviewed in [1,2]). Necrosis is most frequently observed during cell injury, and is closely associated with diseases such as stroke, neurodegeneration, chronic inflammation, and cancer [3–7]. Although necrosis was historically considered an uncontrolled cell death event caused by acute damage, recent discoveries made in multiple organisms demonstrated that in addition to injury-induced necrosis, cells possess genetic pathways that specifically trigger necrosis in response to extracellular or intracellular stimuli (reviewed in [8–11]). For instance, tumor necrosis factor (TNF) induces a necrosis pathway executed through Ser/Thr kinases [10]. In addition, hyperexcitation of neurons or glial cells induced by the massive release of neurotransmitters or constitutively active ion channels cause excitotoxic necrosis [7,12,13]. Unlike apoptosis, which relies on caspase-mediated death-triggering mechanisms, known necrosis-triggering pathways appear to be independent of caspase-activities (reviewed in [8,14]). On the other hand, like apoptotic cells, in many cases necrotic cells have been observed to be engulfed by phagocytes [15,16]. Efficient clearance of necrotic cells from animal bodies helps to resolve the wounded area; furthermore, cell-corpse removal is essential for reducing harmful inflammatory and auto-immune responses induced by the contents of necrotic cells [15,17]. It is currently unclear how necrotic cells expose the “eat me” signal molecules on their surfaces to attract engulfing cells.

**Fig 1:**
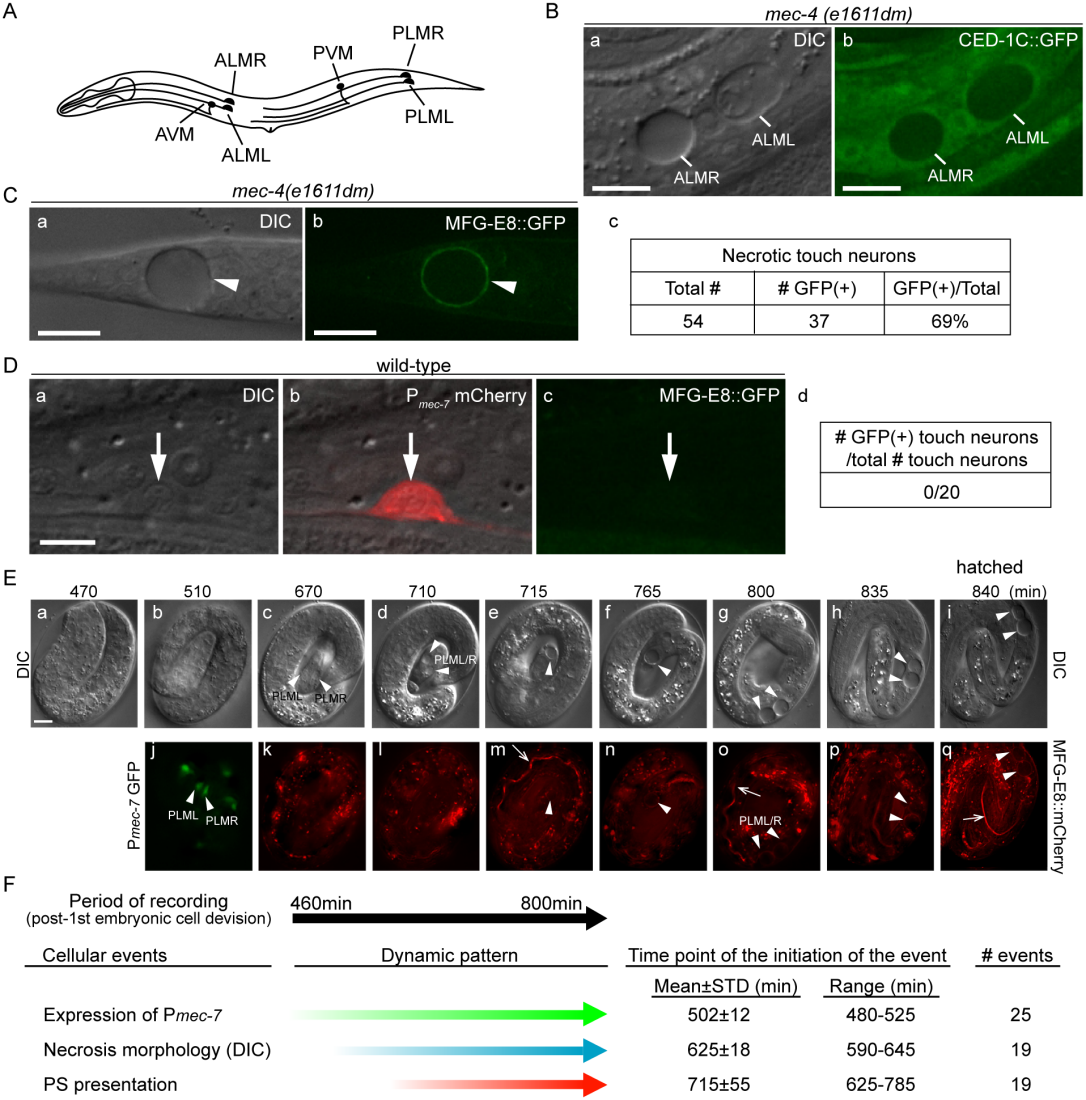
Necrotic cells actively expose PS on their surfaces. (A) The position and name of the six touch neurons in *C*. *elegans*. (B) Necrotic touch neurons are engulfed by hypodermal cells. DIC (a) and GFP (b) images of 2 necrotic touch neurons ALML and ALMR (marked by white lines) engulfed inside larval hypodermis labeled with CED-1C::GFP expressed and distributed in the cytoplasm in hypodermal cells under the control of P_*ced-1*_ in the *mec-4(e1611dm)* genetic background. Scale bars are 10μm. (C) PS is exposed on the surface of necrotic cells. (a) DIC and (b) corresponding epifluorescence images detecting MFG-E8::GFP on the surface of a necrotic touch neuron (white arrowheads) in the tail of a L1 larva. P_*dyn-1*_ *mfg-e8*::*gfp* is expressed in the *mec-4(e1611dm)* background. (c) The number of necrotic touch neurons labeled with MFG-E8::GFP on their surfaces and the total number of necrotic touch neurons analyzed in 30 animals. Dorsal is to the top. Scale bars are 10μm. (D) PS is not detected on the outer surface of live touch neurons. (a) DIC, (b) mCherry/DIC merged, and (c) GFP images of a live touch neuron (arrows) in the tail region of a wild-type L3 larva co-expressing P_*mec-7*_ mCherry (touch neuron marker) and P_*dyn-1*_ MFG-E8::GFP. (d) MFG-E8::GFP is not observed in any of the 20 live touch neurons characterized. Dorsal is to the top. Scale bars are 5μm. (E) Time-lapse images monitoring the appearance of PS on the outer surface of necrotic PLML and PLMR during embryogenesis. The touch neuron-specific reporter construct is P_*mec-7*_ *gfp*. The PS reporter construct is P_*dyn-1*_ *mfg-e8*::*mCherry*. Time points are marked as min post 1^st^ embryonic division. Recording started at the 2-fold stage (460 min) (a) and ended when the embryo hatched (i), in 5-min interval. White arrowheads in (j) indicate living PLML and PLMR. White arrowheads in all other panels indicate the same cells, which are PLMR and/or PLML, that undergo necrosis. The white open arrowhead in (m, o, q) mark intestinal lumen, within which the secreted MGF-E8::mCherry is visible. The scale bar is 5μm. (F) Summary of the quantitative analysis of the time-lapse recording monitoring the dynamics of three features of necrotic PLML and PLMR: (1) the specification of touch neurons (the expression of P_*mec-7*_ GFP), (2) the distinct swelling morphology of necrotic cells observed under DIC microscope, and (3) the appearance of PS on the surface of necrotic cells (MFG-E8::mCherry enrichment on necrotic cells). The time points of event initiation are represented as “min-post the 1st cleavage”.

Besides being an excellent model organism for studying the mechanisms of apoptosis and the removal of apoptotic cells [18], the soil nematode *Caenorhabditis elegans* has also been established as a model for studying necrosis [8,13]. In *C*. *elegans*, a number of mutations in the subunits of ion channels, the acetylcholine receptor, and trimeric GTPases induce specific neurons to undergo necrotic cell death that mimics the excitotoxic necrosis, which occurs during stroke, trauma, and neurodegenerative disorders in humans (reviewed in [8]). In particular, specific mutations in multiple genes trigger the necrosis of six mechanosensory (touch) neurons (AVM, PVM, ALML/R and PLML/R) required to sense gentle mechanical stimuli along the body wall [19–21]. Dominant (*dm*) mutations in *mec-4*, which encodes a core subunit of a multimeric, mechanically gated sodium channel belonging to the DEG/ENaC family specifically expressed in the touch neurons, lead to hyperactive channel conductivity of Na^+^ and Ca^2+^ and induce these neurons to undergo necrosis [19,22]. In *mec-4(dm)* mutants, the six dying neurons swell to many times their original sizes and develop cytoplasmic vacuoles and large membranous whorls, and are easily distinguishable from living or apoptotic cells under Differential Interference Contrast (DIC) optics by their giant sizes (Fig 1) [16,21]. This type of cell death does not require CED-3 caspase activity [23], and is instead triggered by the influx of Ca^2+^ into the cytoplasm [22,24]. Despite their distinct modes of triggering cell death, the seven *ced* genes needed for the engulfment of apoptotic cells are also required for the efficient removal of necrotic touch neurons [25], indicating the presence of certain common recognition and engulfment mechanisms for dying cells. On the other hand, the distinct cellular features observed during macrophage engulfment of necrotic mammalian cells imply that unique pathways exist to clear necrotic and apoptotic cells [26].

Phosphatidylserine (PS), a membrane phospholipid, is a known “eat me” signal presented on the surface of apoptotic cells to directly or indirectly attract phagocytic receptors such as *C*. *elegans* CED-1, *Drosophila* Draper, and mammalian Tim4 and BAI1, leading to the initiation of their engulfment [27–31]. In living cells, PS is almost exclusively localized to the inner leaflet of the plasma membrane, at least partially due to an ATP-dependent aminophospholipid translocase activity that selectively returns PS and PE (phosphatidylethanolamine) from the outer to the inner leaflet [32–34]. During the early stage of apoptosis, PS is detected on the outer leaflet, suggesting a process of trans-bilayer redistribution [32,33]. Phospholipid scramblases, by catalyzing the random, bi-directional “flip-flop” of phospholipids across the membrane bilayer, could potentially counter the aminophospholipid translocase activity [35]. The mouse transmembrane protein 16F (TMEM16F) was recently found to act as a novel Ca^2+^-activated phospholipid scramblase [36]. However, TMEM16F does not seem to be involved in exposing PS on apoptotic cell surfaces [37]. On the other hand, mouse Xk-related protein 8 and CED-8, its *C*. *elegans* homolog, mediate PS exposure in response to apoptotic stimuli [38,39]. These results suggest that different phospholipid scramblases function in different cell types and in response to different stimuli. In addition, the mammalian ATP-binding-cassette transporter A1 (ABCA1) has been implicated in the translocation of PS from the inner to the outer leaflet [40,41], although evidence to the contrary also exists [42].

Previously, using milk-fat-globule EGF8 (MFG-E8::GFP), a GFP-tagged, secreted PS reporter, we have detected the presentation of PS specifically on the surface of apoptotic cells during animal development [43]. We have further identified two alternative mechanisms that promote PS exposure in apoptotic somatic and germ cells, respectively [43]. The PS exposure on apoptotic cell surface during embryonic development, which is necessary for their engulfment, relies on the function of *C*. *elegans* CED-7, a homolog of mammalian ABCA1 transporters [43].

Considering the insights that have been made to understand how apoptotic cells are recognized and removed, the mechanisms by which necrotic cells are engulfed remain poorly defined. In particular, it is unclear whether necrotic cells are capable of the active presentation of “eat me” signaling molecules such as PS to attract engulfing cells. Rather, it was assumed that PS was detected on necrotic cell surfaces due to the rupture of necrotic cell membranes [44]. The work reported here establishes that necrotic *C*. *elegans* touch neurons actively present PS on their outer surfaces while maintaining plasma membrane integrity. It further defines two mechanisms that act in parallel to promote the exposure of PS on necrotic cell surfaces, one that is shared with apoptotic somatic cells, and another that is unique to necrotic touch neurons.

## Results

### Necrotic touch neurons actively externalize phosphatidylserine on their surfaces

The dominant mutant allele *e1611dm* of *C*. *elegans mec-4* results in the necrotic death of six touch neurons, which swell to several times of their original diameter (Fig 1A and 1B(a)), displaying a morphology distinct from somatic apoptotic cells, which undergo cytoplasmic shrinkage and nuclear condensation [19,21,45–47]. Previously, necrotic AVM and PVM were observed inside the hypodermis [16]. To visualize the engulfment status of all necrotic touch neurons, we expressed P_*ced-1*_ *ced-1C*::*gfp*, which produced a GFP reporter that is distributed evenly in the cytoplasm of engulfing cell types, including hypodermal cells [48]. We detected all six necrotic touch neurons as dark holes embedded inside the GFP-labeled engulfing cells in newly hatched *mec-4(e1611dm)* larva (Fig 1B), establishing that necrotic touch neurons are engulfed by hypodermal cells.

To examine whether necrotic cells expose PS on their cell surfaces, we expressed *mfg-e8*::*gfp*, a secreted PS reporter [43], in *mec-4*(*e1611dm*) mutant worms. In newly hatched L1 larvae, we detected GFP specifically enriched on the surface of necrotic touch neurons (Fig 1C). In contrast, when *mfg-e8*::*gfp* was expressed in *mec-4(+)* worms, no fluorescence was detected on the surface of living touch neurons (Fig 1D). These results suggest that PS is present specifically on the surface of cells that undergo necrosis.

Previously, it was generally believed that necrosis caused prominent plasma membrane rupture [1,2]. If that is the case, it is possible that MFG-E8::GFP molecules penetrate through the plasma membrane and associate with the PS molecules on the inner leaflet of the plasma membrane. To distinguish whether the enriched MFG-E8 signal is a result of PS exposure on the outer or inner surfaces of necrotic cells, we examined whether the necrotic touch neurons observed in the *mec-4(e1611dm)* mutants lost plasma membrane integrity. We observed the localization of GFP or mRFP reporters expressed specifically in touch neurons under the control of the *mec-7* promoter (P_*mec-7*_) [49] in *mec-4(e1611dm)* worms and found that the fluorescent signals were exclusively retained inside necrotic cells (S1A (a, b, e, f) Fig). In parallel, secreted GFP (ssGFP) reporters, which are tagged with a signal sequence from SEL-1 [50] and expressed specifically from hypodermal (P_*col-10*_) and body wall muscle (P_*myo-3*_) cells [28,51], two types of cells that neighbor the touch neurons, were not observed inside touch neurons (S1A (c, d, g, h) Fig). These GFP signals were detected inside coelomocytes, mobile cells that possess high endocytic activity (S1B Fig), indicating that they are indeed secreted into the close proximity of the touch neurons. No plasma membrane penetration of the touch-neuron reporter or neighboring-cell reporters was observed during a 36-hr observation period from the appearance of necrotic cell morphology in embryos to the mid-L4 stage. The above lines of evidence indicate that the plasma membrane of necrotic touch cells is not permeable to GFP or mRFP molecules (which are of sizes between 25 and 27 kD). Thus, it is unlikely that the same plasma membrane would be permeable to MFG-E8::GFP, which is substantially larger (78 kD). Furthermore, when wild-type and *mec-4(e1611dm)* worms were stained with propidium iodide, a small molecular weight (MW = 688 Da) fluorescent dye that is not permeable across the intact plasma membrane (Materials and Methods), we did not observe propidium iodide signal in the living or necrotic cells (S1C Fig). The only propidium iodide signal observed came from the intestinal track, inside which were ingested propidium iodide-stained bacteria cells (S1C Fig). Together, the above results indicate that, against the common belief that necrotic cells passively expose PS through plasma membrane rupture and in this manner attract engulfing cells, the *C*. *elegans* necrotic touch neurons maintain cell integrity and actively expose PS, which may function as a specific “eat me” signal, on their surfaces.

Using a live-cell recording protocol that we established for touch neurons (Materials and Methods), we monitored the dynamics of an MFG-E8::mCherry reporter during embryogenesis. Among the six touch neurons, four are born during mid embryogenesis, including PLML and PLMR, which were reported to arise at approximately 510 min post the 1^st^ embryonic cell division (the 1^st^-cleavage), whereas AVM and PVM were reported to be born at the L1 larval stage [46,47]. At hatching, PLML and PLMR should have existed for 290 min. We observed that the enrichment of PS on necrotic PLML and PLMR was a gradual process after necrosis was initiated at a morphological level (Fig 1E and 1F) (S1 Movie). Among the following three events, (1) the differentiation of touch neurons, which is indicated by the expression of P_*mec-7*_ *gfp*, (2) the swelling of touch neurons undergoing necrosis, which is visible under DIC optics, and (3) the exposure of PS on the outer surface, indicated by the enrichment of MFG-E8::mCherry, cell differentiation occurs the earliest, initiating approximately 480–525 min after the 1^st^ cleavage (Fig 1E(b, j) and 1F) (S1 Movie). On average 120 min later, the swelling morphology of PLML/R starts to develop, indicating that the constitutively active Na^+^/Ca^2+^ channel starts to initiate necrosis. The first time point when the enrichment of PS is detected on PLML/R surface varies; yet in all 19 cases monitored, it occurs after the initial appearance of the necrotic morphology observed by DIC (S1 Movie). Subsequently, the PLML/R surface MFG-E8::mCherry intensity continues to increase until the time point of hatching (Fig 1E(p and q)). Our observations established the order of the initiation of these three events, and further suggest a causal relationship between the initiation of necrosis and the exposure of PS.

### Multiple types of necrotic neurons expose PS on their surfaces

To determine whether PS-externalization is a general phenomenon occurring to different types of neurons that undergo necrosis, we monitored PS enrichment on non-touch neurons. *u662*, a gain-of-function mutation in *deg-3*, which encodes a subunit of an acetylcholine receptor ion channel [20], causes the necrosis of the six touch neurons and a few additional sensory and inter-neurons through hyper-activation of the acetylcholine receptor ion channel [20]. Cells undergoing necrosis in *mec-4* and *deg-3* dominant mutants display the same distinct morphology [19,20,52,53]. In *deg-3(u662)* animals, we detected PS on the surface of necrotic neurons, including touch neurons and other types of neurons (S2 Fig). This result suggests that PS-exposure is a general feature of neurons induced to undergo necrosis through excitotoxicity.

### CED-1 recognizes necrotic cells and initiates necrotic-cell engulfment

**Fig 2:**
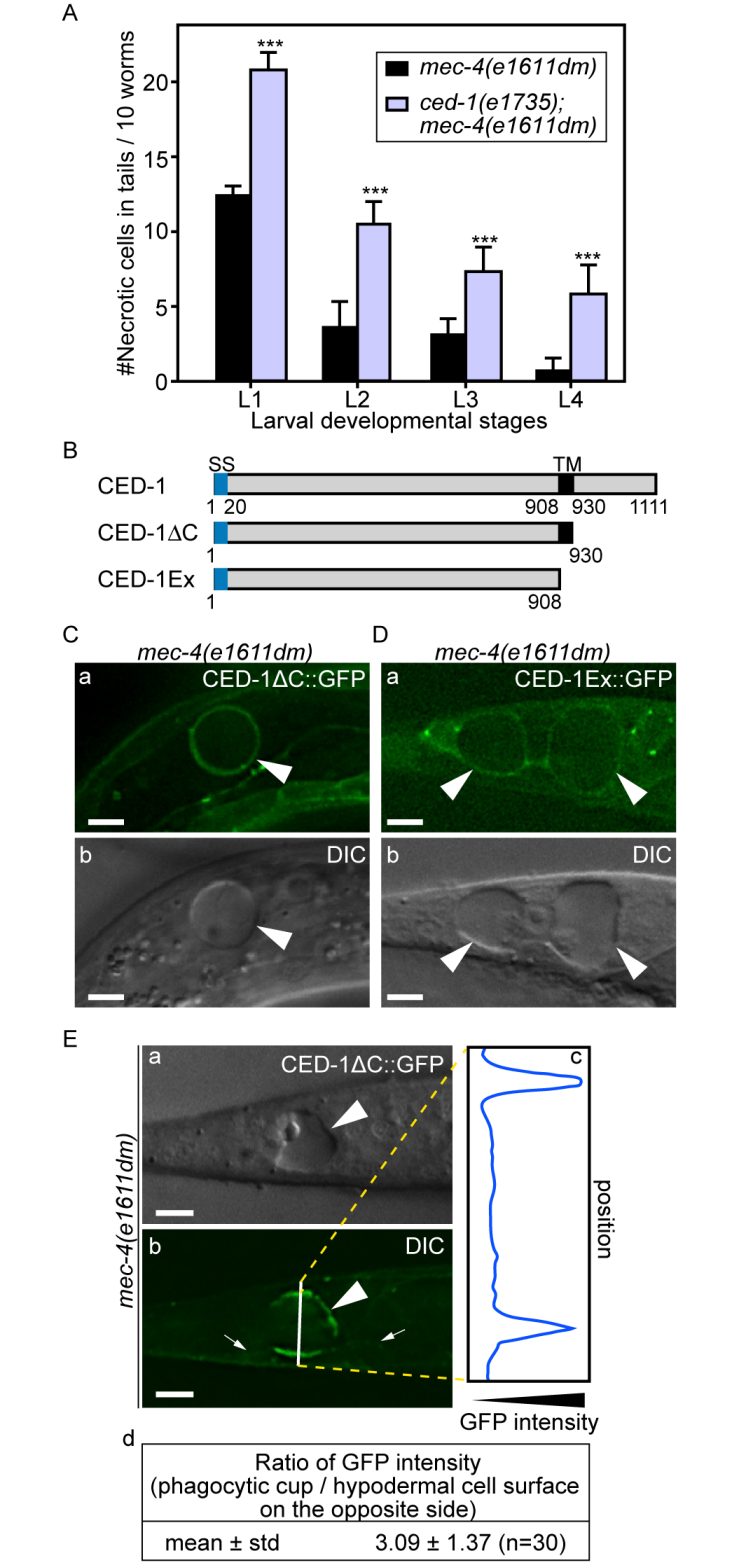
Necrotic cells are recognized by phagocytic receptor CED-1. (A) Inactivating *ced-1* perturbs the removal of necrotic cells. Worms of indicated genotypes in four larvae stages were scored for the number of necrotic corpses in the tail. For each data point, 3 groups of 20 animals per group were scored. Data are presented as mean ± sd. ***, p<0.001, Student *t*-test. (B) Domain structures of CED-1 and its truncated forms. Numbers indicate the beginning and ending residues of each domain. SS, signal sequence. TM: transmembrane domain. (C-D) CED-1ΔC::GFP (C) and CED-1Ex::GFP (D) expressed in engulfing cells cluster around necrotic cells. Epifluorescence (a) and corresponding DIC (b) images of CED-1ΔC::GFP(C) or CED-1Ex::GFP (D) expressed in hypodermal cells under the control of P_*ced-1*_ in the *mec-4(e1611dm)* background. Arrowheads mark necrotic cells on which GFP clusters around. Dorsal is to the top. Scale bar are 5μm. (E) Quantitative comparison between the signal intensity of CED-1ΔC::GFP on a phagocytic cup (arrowhead) engulfing an necrotic cell (arrowheads) and on the side (small arrows) of the engulfing cell opposite to the phagocytic cup. (a-b) Epifluorescence (a) and corresponding DIC (b) images of the tail of an L1-stage larva expressing CED-1ΔC::GFP in the hypodermal cells. One white vertical line in (b) is where quantitative line profile of GFP intensity shown in (c) is generated. Dorsal is to the top. Scale bars are 6μm. (c) The GFP signal intensity line profile corresponds to the white line in (b) drawn across the engulfing hypodermal cell, reaching each side of the worm’s tail. (d) For each of the thirty phagocytic cups or phagosomes containing necrotic cells, signal intensity of three random points on the surface of phagocytic cups or phagosomes were measured and averaged, so were that of three points on the side of the engulfing cell opposite to the necrotic cell being engulfed. The mean ratio of the two sets of signal intensity data and the standard deviation (std) were shown.

CED-1 is a phagocytic receptor that is localized on the surface of several types of cells, including all engulfing cell types and clusters around apoptotic cells in response to the neighboring “eat me” signals [28,43]. To determine the efficiency of necrotic cell clearance in the *ced-1(e1735); mec-4(e1611dm)* double mutants, we chose to score the dynamic presence of necrotic PLML and PLMR, two touch neurons in the tail that undergo necrosis during mid-embryogenesis, throughout all larval stages. The rationale is that the longer a necrotic touch cell persists during larval development, the less efficient its removal process must be. The same scheme was used to score the efficiency of necrotic cell clearance throughout this report (Materials and Methods). We found the *ced-1(e1735)* null mutation greatly reduced the efficiency of necrotic cell removal (Fig 2A), consistent with a previous report [25]. Prior results indicate that the extracellular domain of CED-1 is responsible for recognizing the surface feature(s) of apoptotic cells. We analyzed two truncated forms of CED-1 for their ability to recognize necrotic cells by monitoring GFP-tagged truncated forms expressed in larval hypodermal cells, which engulf necrotic touch neurons. We first found that CED-1ΔC::GFP, a truncated form of CED-1 (Fig 2B) that remains bound to the plasma membrane of the engulfing cells, is highly enriched on the phagocytic cup or phagosomal surface (Fig 2C and 2E) in comparison to other regions of the plasma membrane of the same cell. Quantification of GFP fluorescence intensity on phagocytic cups or phagosomes is on average 3.1 times of that on other plasma membrane regions of the same cell (Fig 2E(d)). We next found that CED-1Ex::GFP, a truncated and secreted form of CED-1 (Fig 2B), was specifically enriched on the surfaces of necrotic cells (Fig 2D). These results indicate that the extracellular domain of CED-1 directly recognizes necrotic cells, and that the high level of CED-1ΔC::GFP detected on the engulfing cell membrane region around necrotic cells is not merely a result of necrotic touch neurons being embedded inside hypodermal cells.

### The extracellular domain of CED-1 directly associates with PS

**Fig 3:**
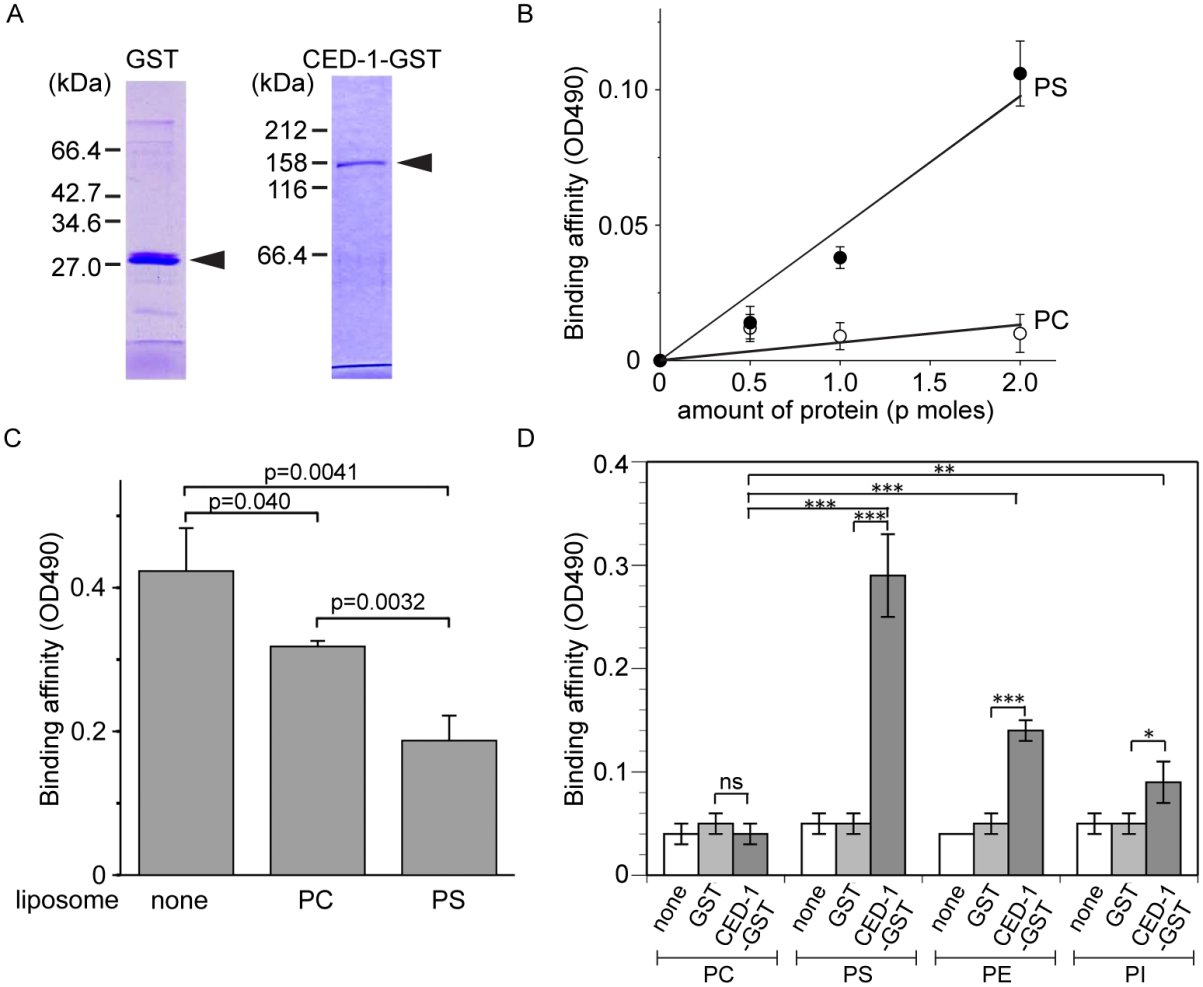
The extracellular domain of CED-1 directly associates with PS. The entire extracellular region of CED-1 fused to GST (CED-1-GST) was tested for the binding to phospholipids. Data were representative of three independent experiments that yielded similar results. (A) Detecting the purified GST or CED-1-GST using SDS-PAGE. Molecular weight markers are marked on the side. Arrowheads mark the purified proteins. (B) The binding affinities of identical amount of CED-1-GST and GST to PS and PC were measured in ELISA-like reactions. Data shown here are results after subtracting the OD value corresponding to GST from that corresponds to CED-1-GST at each data point. (C) The binding affinity of CED-1-GST (6.3 p mole in 0.1ml volume) to PS was analyzed in ELISA-like reactions in the presence of PC-only liposomes (0.5 mM) or PS-containing liposomes (0.5 mM) as competitors. (D) The binding of GST and CED-1-GST (both in 6.3 pmol in 0.1ml volume) to various phospholipids, including PC, PS, phosphatidylethanolamine (PE) and phosphatidylinositol (PI), was examined in ELISA-like reactions. *, 0.002<*p*<0.005; **, 0.001<*p*<0.002; ***, *p*<0.001; ns, no significant difference; Student *t-*test.

Since necrotic cells, like apoptotic cells, specifically expose PS on their surfaces, and because CED-1 recognizes both necrotic and apoptotic cells *in vivo*, we tested whether PS could act as a ligand for CED-1. The extracellular domain of CED-1 was expressed as a fusion protein to glutathione *S*-transferase (GST) (CED-1-GST) in an insect cell expression system, affinity purified by glutathione-sepharose chromatography (Fig 3A), and tested for its binding affinities to PS *in vitro* (Materials and Methods). We first employed an Enzyme-Linked ImmunoSorbent Assay (ELISA)-like reaction to test the interaction between CED-1-GST and PS or phosphatidylcholine (PC). The CED-1 protein displayed efficient association with PS but not PC, in a dose-dependent manner (Fig 3B). We next examined this association in a surface plasmon resonance assay [29]. In this assay, CED-1-GST was applied onto channels of the HPA chip, on which equivalent numbers of PS-containing liposomes and PC-only liposomes had been immobilized (S3 Fig). We found that the values in response units after the injection of CED-1-GST to the channel with PS-containing liposome were higher than those obtained for the control channel (S3B Fig). These two assays indicate that the extracellular domain of CED-1 interacts directly with PS. Furthermore, free PS-containing liposomes, but not PC-liposomes, could efficiently compete with PS-containing liposomes coated on the well for binding to CED-1-GST in the ELISA-like reactions (Fig 3C), suggesting that CED-1 specifically recognizes PS as a component of membrane bilayer. In the ELISA-like assay, binding between CED-1-GST with two other phospholipids, phosphatidylethanolamine (PE) and phosphatidylinositol (PI), was also detected (Fig 3D). All these results indicate that the extracellular domain of CED-1 directly associates with phospholipids containing an amino group or negative charge, including PS, and support the hypothesis that PS serves as a ligand for CED-1 for the recognition of necrotic and apoptotic cells.

### CED-7 is essential for PS exposure on the surface of necrotic cells

**Fig 4:**
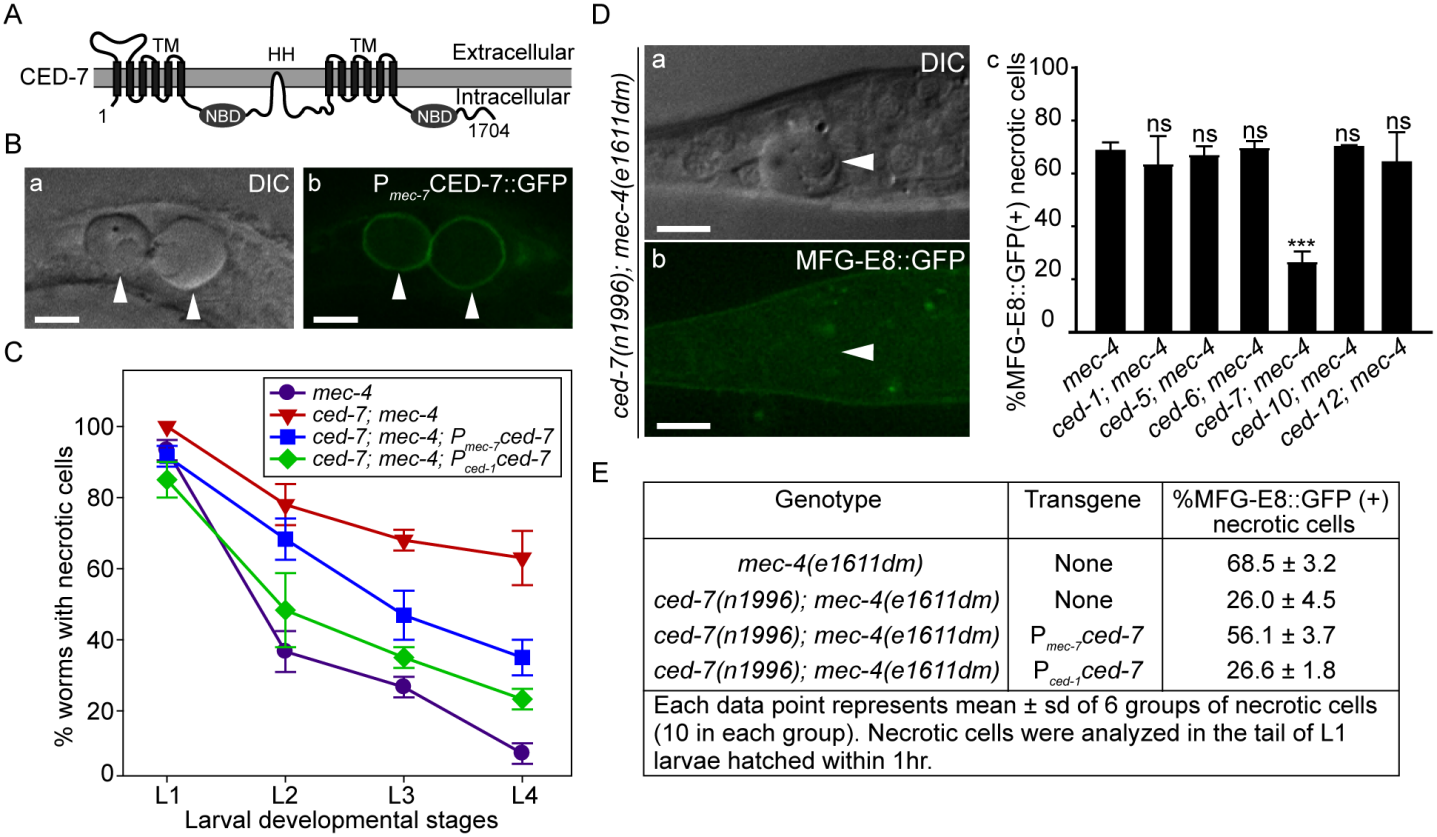
CED-7 promotes necrotic cell removal by acting in two different cell types. (A) CED-7 domain diagram. TM: transmembrane region (each has 6 transmembrane domains). HH: highly hydrophobic motif. NBD: nucleotide-binding domain. (B) CED-7::GFP expressed in necrotic cells is localized to cell surface. DIC (a) and corresponding epifluorescence (b) images of the tail of a newly hatched *mec-4(e1611dm)* L1 larva (hatched within 1hr) expressing P_*mec-7*_ *ced-7*::*gfp*. Arrowheads mark two CED-7::GFP-positive necrotic corpses. Dorsal is to the top. Scale bars are 5μm. (C) Expression of *ced-7* cDNA either in touch neurons or engulfing cells each partially rescues the necrotic cell removal defects. *ced-7(n1996)*; *mec-4(e1611dm)* larvae expressing either P_*ced-1*_ *ced-7* or P_*mec-7*_ *ced-7* were scored for the number of necrotic corpses in indicated larvae stages. Each data point represents the mean of 3 groups of 20 animals per group. Error bars represent sd. (D) CED-7 is required for PS exposure. (a) DIC and (b) the corresponding epifluorescence images of a necrotic cell (arrowheads) lacking MFG-E8::GFP signal on its surface in the tail of a L1 larva hatched within 1hr. Dorsal is to the top. Scale bar: 5μm. (c) Bar graph showing the percentage of necrotic cells on which the PS is detected by MFG-E8::GFP in newly hatched L1 larvae in different engulfment mutant backgrounds. Alleles used here: *mec-4(e1611dm)*, *ced-1(e1735)*, *ced-6(n2095)*, *ced-7(n1996)*, and *ced-10(n1993)*. Each data point represents the mean percentage calculated from 6 groups of necrotic cells (10 in each group). Error bars represent sd. ns: no significant difference (p>0.05); ***, p<0.001, Student *t*-test. (E) The function of CED-7 in necrotic cells specifically rescues the PS-exposure defect of *ced-7* mutants. The percent of necrotic cell corpses labeled by MFG-E8::GFP were scored in *ced-7; mec-4* mutants expressing each transgene of P_*mec-7*_ *ced-7* or P_*ced-1*_ *ced-7* individually.

Among the seven engulfment *ced* genes that act in two parallel pathways, *ced-7*, which encodes a member of the ABC transporter family (Fig 4A), is the only one required for the presentation of PS on the outer surface of apoptotic cells in *C*. *elegans* embryos [43]. The *ced-7(n1996)* null mutation severely impairs the removal of necrotic touch neurons (Fig 4C). We found that among the null or strong loss-of-function mutations of six *ced* genes, only the *ced-7(n1996)* mutation significantly reduced the percentage of necrotic touch neurons exhibiting surface PS (Fig 4D). These results indicate that CED-7 is essential for PS exposure on necrotic touch neurons. In support of this conclusion, a CED-7::GFP reporter expressed in touch neurons (P_*mec-7*_ *ced-7*::*gfp*) is observed on the surface of necrotic touch neurons in *mec-4(e1611dm)* background (Fig 4B), consistent with a role of CED-7 in PS-flipping from one leaflet of the plasma membrane to the other.

CED-7 is broadly expressed in all cells [54]. To determine whether *ced-7* functions to promote necrotic cell removal in the engulfing cells or in touch neurons, we tested the effect of cell type-specific expression of *ced-7* in the rescue of *ced-7* mutant phenotypes. The *ced-7(n1996)* null mutation severely delays necrotic cell removal, resulting in the persistent existence of necrotic touch neurons in more than 60% of L4 larvae (Fig 4C). Expression of *ced-7* in either touch neurons (P_*mec-7*_ *ced-7*) or neighboring engulfing cells (P_*ced-1*_ *ced-7*) each partially rescued the necrotic-cell removal defect (Fig 4C), indicating that the functions of CED-7 in necrotic and engulfing cells both contribute to the efficient removal of necrotic cells. We further found that the specific expression of *ced-7* in touch neurons but not in neighboring engulfing cells primarily rescued the PS exposure defect of *ced-7(n1996)* mutants (Fig 4E). This result clearly indicates that the touch cell-specific role of CED-7 is responsible for promoting PS exposure.

### ANOH-1, the *C*. *elegans* homolog of mammalian TMEM16F plays a specific role for the exposure of PS on the surfaces of necrotic cells

**Fig 5:**
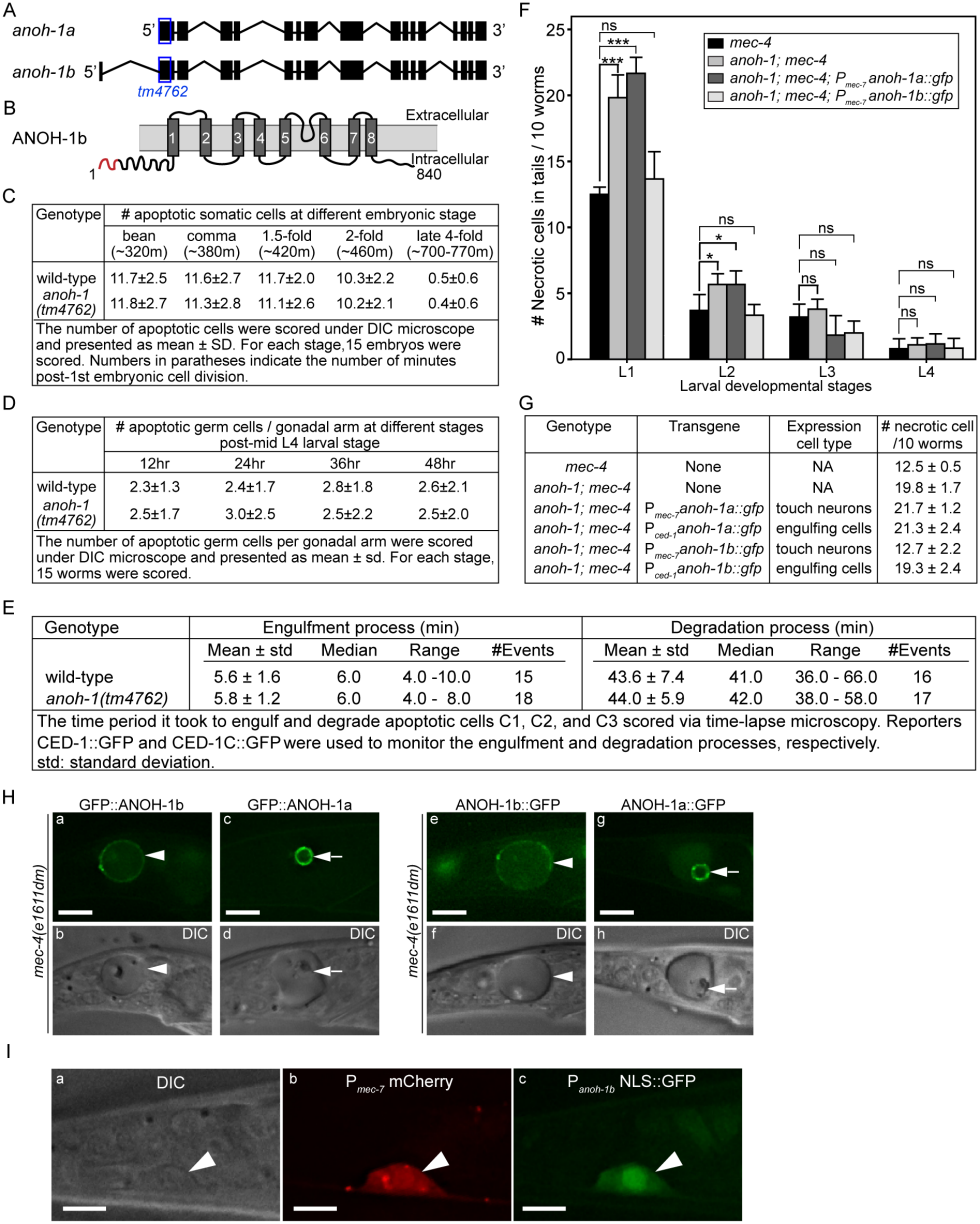
*anoh-1* is required for the efficient removal of necrotic touch cells but not apoptotic cells. (A) Gene structure of two isoforms of *anoh-1*. The blue open boxes indicate the region deleted in the *anoh-1(tm4762)* allele. (B) Domain structure of ANOH-1b protein. Dark grey bars indicate 8 predicted transmembrane domains. Red color labels amino acids 1–18, which exists in ANOH-1b but missing from ANOH-1a. (C) The *anoh-1(tm4762)* mutant embryos are normal in the removal of somatic apoptotic cells. (D) The *anoh-1(tm4762)* mutant adults are normal in the removal of apoptotic germ cells. (E) The engulfment and degradation processes of embryonic apoptotic cells C1, C2, and C3 are normal in *anoh-1(tm4762)* mutant embryos, measured by time-lapse microscopy. (F) The *anoh-1(tm4762)* mutation perturbs the removal of necrotic cells, and the *anoh-1b* but not the *anoh-1a* form rescues the necrotic cell-removal defect when expressed in touch cells under the control of P_*mec-7*_. For each sample, 6 groups of 10 worms at indicated stages were scored and displayed as mean. Error bars indicate standard deviations. *, 0.01<p<0.05 (student *t*-test). ***, p<0.001 (student *t*-test). ns, no significant difference, p>0.05 (student *t*-test). (G) The expression of *anoh-1b* in the necrotic cells, not engulfing cells, rescues *anoh-1(tm4762)* mutant phenotype. In *anoh-1(tm4762); mec-4(e1611dm)* mutant animals expressing indicated transgenes, the number of necrotic cells in the tail of each of the 6 groups of 10 newly hatched L1 larvae were scored. Data are presented as mean ± sd. (H) The GFP fusion forms of ANOH-1b but not ANOH-1a are localized to the plasma membrane. GFP (a, c, e, g) and DIC (b, d, f, h) images of the tails of *mec-4(e1611dm)* L1-stage larvae. Arrowheads in (a, b, e, f) label necrotic cells on which ANOH-1b is observed on the cell surface. Arrows in (c, d, g, h) label necrotic cells in which ANOH-1a is observed on the nuclear surface. All GFP reporters are expressed under the P_*mec-7*_ promoter control. (I) The *anoh-1b* promoter is expressed in touch neurons. Shown here are epifluorescence (b-c) and the corresponding DIC (a) images of the tail region of a wild-type L2 larva co-expressing P_*mec-7*_ mCherry (b), which is a touch neuron-specific reporter, and P_*anoh-1b*_ NLS::GFP (c). White arrowheads mark a touch neuron. Dorsal is up. Scale bars are 5μm.

Mammalian TMEM16F, a multispan transmembrane domain protein, is a Ca^2+^-activated phospholipid scramblase that triggers PS exposure in response to Ca^2+^-influx [36]. Given that the *mec-4(dm)*-induced touch neuron necrosis is mediated by Ca^2+^ influx [22,24], we examined whether ANOH-1, a close *C*. *elegans* homolog of TMEM16F (Figs 5B and S4), mediated PS exposure when necrosis occurred. We analyzed an *anoh-1(tm4762)* deletion allele (www.wormbase.org) for the removal of dying cells. The *tm4762* allele carries a 202-bp deletion that results in a frameshift and a premature stop codon after amino acid 17 of the predicted ANOH-1b open reading frame and removes the start codon of the alternatively-spliced ANOH-1a open reading frame (S4 and S5B(b) Figs) (also see the next section), presumably generating a null allele. In *anoh-1(tm4762)* mutant embryos, the numbers of apoptotic cells are the same as that displayed in wild-type embryos at 5 different embryonic developmental stages (Fig 5C). Furthermore, the dynamics of the engulfment and degradation processes of three individual apoptotic cells, C1, C2, and C3, are normal comparing to wild-type embryos (Fig 5E), using previously established protocols [48,55]. Similarly, the number of apoptotic germ cells are virtually the same in wild-type and *anoh-1(tm4762)* mutant adult hermaphrodites at four time points (Fig 5D). These results indicate that *anoh-1*, unlike *ced-7*, is not involved in the removal of apoptotic cells. In contrast, in *anoh-1(tm4762)*; *mec-4(e1611dm)* double mutant animals, the removal of necrotic touch neurons is siginificantly delayed: at L1 and L2 stages, the mean numbers of persistent necrotic PLML/R in *anoh-1(tm4762)* background are approximately 1.6-fold and 1.5-fold of that in wild-type larvae, respectively, whereas at L3 and L4 stages, the mean numbers are not significantly different from wild-type animals (Fig 5F). These results strongly suggest that *anoh-1* specifically contributes to efficient removal of necrotic but not apoptotic cells.

**Fig 6:**
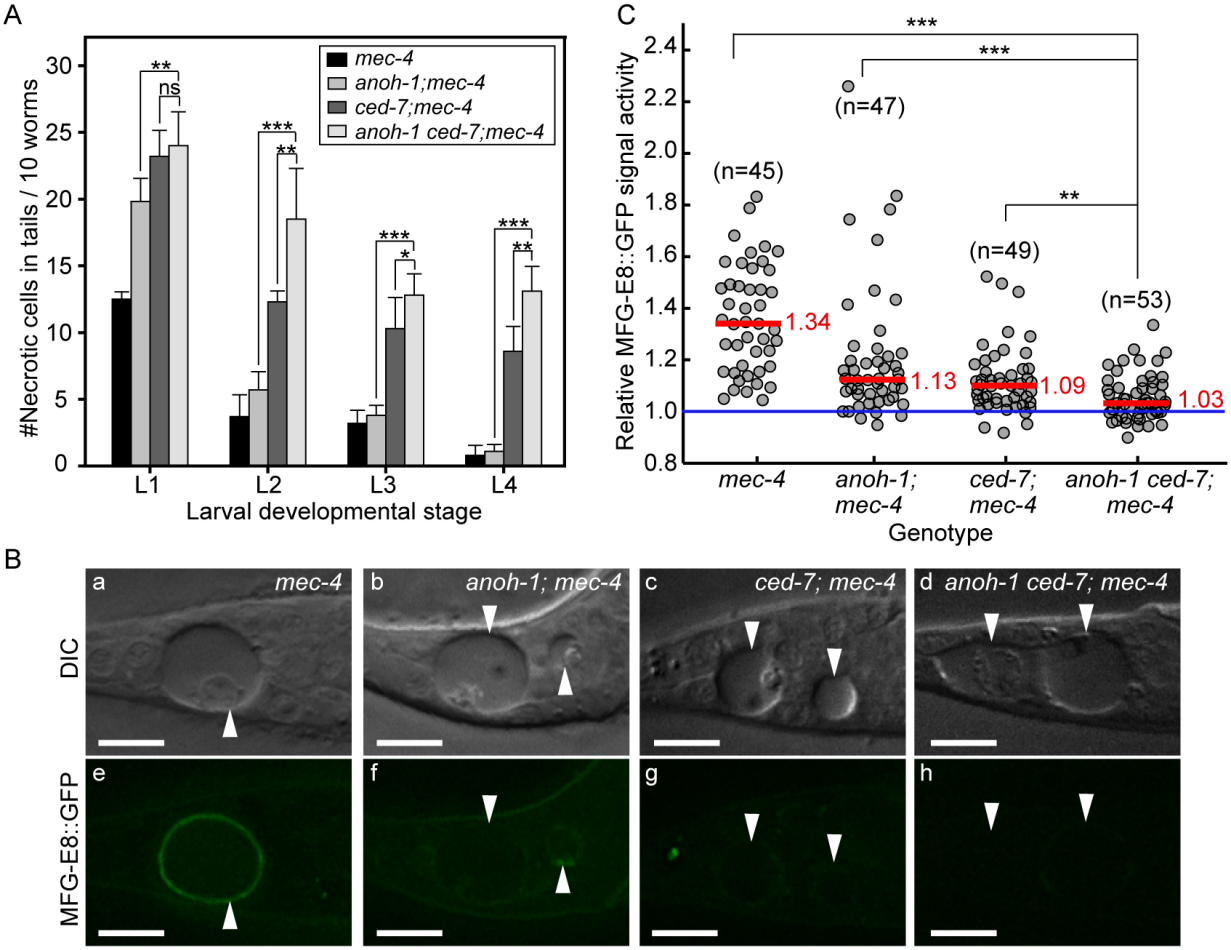
Inactivating both *ced-7* and *anoh-1* results in enhanced phenotypes. Alleles used: *anoh-1(tm4762)*, *ced-7(n1996)*, *mec-4(e1611dm)*. (A) Double mutants display persistent necrotic cells more frequently than each of the single mutants. Mutants of indicated genotypes were scored at four larval stages for the persistence of necrotic corpses in their tails. Sixty animals of each genotype were scored in six groups of 10 worms. Error bars indicate standard deviations of each data point. “***”, “*”, and “ns”, p<0.001, 0.01<p<0.05, and p>0.05, respectively, student *t-*test. (B) ANOH-1 and CED-7 are both needed for presenting PS to the necrotic cell surfaces. DIC (a-d) and corresponding epifluorescence (e-h) images of the tails of newly (within 1hr) hatched L1 larvae expressing P_*dyn-1*_ *mfg-E8*::*gfp* showing different GFP signal on necrotic cell surfaces. White arrowheads label necrotic corpses. Dorsal is up. Scale bars are 10μm. (C) Relative signal intensity of MFG-E8::GFP was calculated as the ratio between GFP signal intensity on surfaces of necrotic cells in the tail and that in a nearby region inside the same worm. Signal intensity measurement was performed using L1 larvae aged within 1-hr of hatching. Each grey circle represents one necrotic cell analyzed. “n” indicates the number of necrotic cells analyzed for each genotype. Red lines represent the median value of each group of sample. “***” and “**”, p<0.001 and 0.001<p<0.01, respectively, student *t-*test.

In wild-type and *anoh-1* mutant animals, we further measured the MFG-E8::GFP signal intensity and calculated the ratio of GFP intensity on necrotic PLML/R to that in an equivalent area of the neighboring live cells (Materials and Methods). Lack of PS enrichment on the surface of necrotic cells will result in a ratio of approximately 1.0. We first quantified the PS signal intensity at the early L1 stage, when the necrotic cell removal defect displayed by the *anoh-1* mutants was the most prominent among all four larval stages (Fig 5F). The *anoh-1(tm4762)* mutation significantly inhibits PS enrichment on necrotic touch neurons, reducing the median value of this ratio from 1.34 in the wild-type animals to 1.13 (Fig 6B and 6C). This result indicates a unique function of ANOH-1 in promoting PS exposure on necrotic cell surfaces. At the young L2 larval stage (15–16 hrs after hatching), the average relative PS signal intensity value increases from the young L1 value in both the wild-type (from 1.36 to 1.71) and *anoh-1(tm4762)* mutant (1.20 to 1.51) strains (S6B Fig), probably as a result of the continuous accumulation of the GFP signal on the surface of necrotic neurons,. The PS signal increase in *anoh-1* mutants could explain the reduced removal defect at later developmental stages (Fig 6A).

### ANOH-1 acts in touch neurons to promote the removal of necrotic touch neurons

Based on its gene structure, *anoh-1* is predicted to encode two splice variants, *anoh-1a* and *anoh-1b* (Fig 5A). The predicted ANOH-1b protein carries an additional 18 residues at the amino-terminus, as compared to the predicted ANOH-1a protein (S4 Fig). Using RT-PCR (Materials and Methods), we detected the existence of the *anoh-1b* transcript in whole worm extracts (S5B Fig). To determine which of the two splice variants and in which cell type *anoh-1* is functional in the removal of necrotic cells, we individually expressed *anoh-1a* and *anoh-1b* in touch neurons (P_*mec-7*_) or engulfing cells (P_*ced-1*_), in the *anoh-1(tm4762)*; *mec-4(e1611dm)* background (Fig 5F and 5G). To monitor the subcellular localization of each isoform, *anoh-1a* and *anoh-1b* were each tagged with *gfp*. Among the four expression constructs tested, only *anoh-1b*, when expressed in touch neurons (P_*mec-7*_ *anoh-1b*::*gfp*), rescued the necrotic cell removal defect of these mutants (Fig 5F and 5G). In addition, P_*mec-7*_ *anoh-1b*::*gfp* also leads to the recovery of PS exposure on necrotic neuron surfaces (S7 Fig). These results indicate that *anoh-1b* is the functional form and that it acts in necrotic touch neurons to promote their removal.

To understand the function of the amino-terminal 18 amino acids present in ANOH-1b, which are absent from the predicted ANOH-1a protein, we characterized the subcellular localization of each form, as N- or C-terminal GFP-tagged proteins, expressed in touch neurons. GFP::ANOH-1b and ANOH-1b::GFP are both localized to the plasma membrane, consistent with the hypothesized role of ANOH-1 in promoting PS on cell surface (Fig 5H(a, b, e, and f)). In contrast, GFP::ANOH-1a and ANOH-1a::GFP are both observed inside touch neurons, enriched on the nuclear surface (Fig 5H(c, d, g, and h)). These results suggest that the plasma membrane localization is essential for the function of ANOH-1 in necrotic cell-removal.

To determine the expression pattern of *anoh-1b*, we cloned the 617 bp DNA fragment immediately 5’ to Exon 1 of *anoh-1b*, and tentatively regard this fragment as the P_*anoh-1b*_ promoter. The nuclear localization sequence (NLS)-tagged GFP signal produced by P_*anoh-1b*_ is expressed in touch neurons (Figs 5I and S5C(l, o, r)). This result supports the touch neuron-specific function of ANOH-1 (the b isoform) in promoting PS-exposure. In addition, by comparing the expression patterns of P_*anoh-1b*_ NLS-GFP and a pan-neuronal expression reporter P_*rab-3*_ dsRed [56], we observed strong P_*anoh-1b*_ activity in many neurons in the head and tail (S5C Fig). This result is consistent with a previous report detecting *anoh-1* expression in sensory neurons [57]. In addition, *anoh-1* expression is also observed in intestinal cells as previously reported [57] and pharyngeal muscles (S5C (g, h, i) Fig).

### ANOH-1 acts in a parallel pathway to CED-7 to regulate PS exposure

To investigate the functional relationship between CED-7 and ANOH-1 in the clearance of necrotic cells, we first compared the numbers of persistent necrotic cells throughout larval developmental in *anoh-1(tm4762) ced-7(n1996)* double mutants and in *anoh-1* or *ced-7* single mutant animals, in the *mec-4(e1611dm)* mutant background. We observed that inactivating *ced-7* causes a stronger necrotic cell removal defect than that of *anoh-1* (Fig 6A). Furthermore, inactivating both *ced-7* and *anoh-1* results in an enhanced necrotic cell removal defect starting at the L2 stage (Fig 6A), suggesting that CED-7 and ANOH-1 might perform partially parallel functions during removal. We further quantified the PS signal intensity at the early L1 stage. Inactivating both *ced-7* and *anoh-1* further reduces the PS signal on the surface of necrotic PLML/R, which is already significantly reduced by the *ced-7* or *anoh-1* single mutations compared to the wild-type background (Fig 6B and 6C) or the *ced-6* mutation, which delays necrotic cell removal but does not affect PS exposure (S8 Fig). The above results suggest that CED-7 and ANOH-1 both contribute to the PS-externalization activity; furthermore, they may do so through partially parallel pathways. At the L2 stage, when the necrotic corpse-removal and PS-exposure phenotypes of *anoh-1* mutants are greatly reduced, the PS-exposure phenotype of the *anoh-1 ced-7* double mutants was similar to that of *ced-7* single mutants (S6 Fig), again suggesting that inactivating *anoh-1* delays but does not block necrotic-cell removal.

### CED-8 makes a minor contribution to the removal of necrotic touch neurons

**Fig 7:**
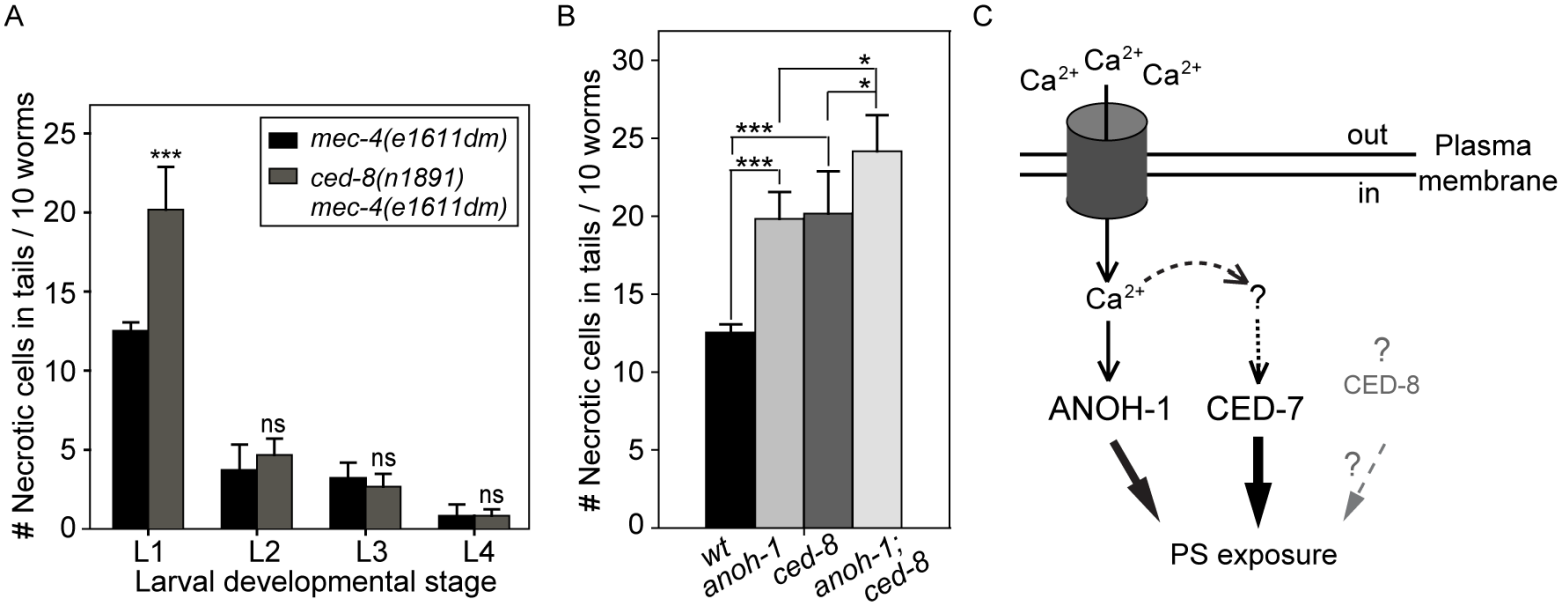
CED-8 contributes to necrotic-cell removal only at the L1 larval stage. (A) The *ced-8(n1891)* mutation perturbs the removal of necrotic touch neurons at the L1 stage. The number of necrotic cells was scored in the tail of worms at four larvae stages. For each data point, 6 groups of 10 animals per group were scored. Data are presented as mean ± sd. ***, p<0.001, ns: no significant difference (p>0.05), student *t*-test. (B) *anoh-1(tm4762); ced-8(n1891)* double mutants display an enhanced necrotic cell removal defect at L1 larval stage as compared to each single mutant. All strains also carry the *mec-4(e1611dm)* mutant allele. Mutants of indicated genotypes were scored at the L1 larval stages for the persistence of necrotic corpses in their tails. Sixty animals of each genotype were scored in six groups of 10 worms. Error bars indicate standard deviations of each data point. “***” and “*”, p<0.001 and 0.01<p<0.05, respectively, student *t-*test. (C) Diagram depicting the hypothesized mechanisms of PS exposure in a neuron undergoing Ca^2+^-induced necrosis. See text for detailed explanation of the models. The dark cylinder represents a multimeric, mechanically gated Na^+^/Ca^2+^ channel, in which MEC-4 is a subunit. This channel is made constitutively open by a dominant mutation in MEC-4. The thickness of the solid lines bearing solid arrows represents the relative contribution of each of the three proteins to the overall PS exposure activity. The solid line bearing an open arrow indicates activation of ANOH-1 by Ca^2+^. The curvy dashed line bearing open arrows indicate Ca^2+^ might activate CED-7. The straight dashed line bearing open arrows and the question marks together indicate that there might exist other upstream activating factors for CED-7 in necrotic cells. Although CED-8 participates in necrotic cell-removal, whether it acts to facilitate the PS-exposure is unknown (represented by the dashed straight line bearing a solid arrow).

We examined whether a loss-of-function mutation of ced-8, which is proposed to encode a phospholipid scramblase essential for PS-exposure on the surface of apoptotic cells, also affects the removal of necrotic touch neurons. In a *ced-8(n1891)* mutant allele, a strong loss-of-function allele that carries a nonsense mutation, truncating CED-8 (458aa) after aa 219 [58], there is a significant necrotic touch neuron removal defect at the L1 larval stage (Fig 7A). However, this defect was not observed in any of the later larval stages (Fig 7A), suggesting that CED-8 merely delayed but did not block necrotic cell removal. We further analyzed the functional relationship between *anoh-1* and *ced-8*. At the L1 stage, the *anoh-1(tm4762); ced-8(n1891)* double mutants display a more severe necrotic cell-removal defect as compared to each single mutant (Fig 7B). These results suggest that *anoh-1* and *ced-8* act in parallel and thus the necrotic cell removal function of *ced-8* is independent of *anoh-1*.

## Discussion

### Active externalization of PS on the surface of necrotic cells

The presentation and function of PS on the surface of cells undergoing necrosis have previously been overlooked, primarily because of the long-existing notion that these cells encounter injury and lose plasma membrane integrity [44,59,60]. However, in recent years, accumulating evidence has demonstrated that in addition to cell injury, necrosis could also be induced by genetic programs (reviewed in [9]). In short, multiple molecular mechanisms exist that induce and execute necrosis [9,14]; thus all necrotic cells do not necessarily lose plasma membrane integrity. The loss of membrane integrity of necrotic cells in culture, where there are no phagocytes to engulf them, does not necessarily represent what happens inside animal bodies, where engulfing cells target dying cells at early stages of their death [16,61] (our own observations). On the other hand, those necrosis events that occur inside animal bodies were rarely examined for plasma membrane integrity. In the case of neuronal excitotoxic necrosis, electron microscopic studies of rat brains and *C*. *elegans* touch neurons reported the swelling of necrotic cells and the presence of surrounding phagocytes, yet no loss of plasma membrane integrity [16,62]. By monitoring signals elicited from GFP or mRFP reporters either inside or outside necrotic cells and by incubating worms with propidium iodide, a small molecule dye that is not plasma membrane permeable, we observed that the necrotic *C*. *elegans* touch neurons induced by excitotoxicity maintain plasma membrane integrity throughout embryonic and larval development. These results indicate that the common notion that necrotic cells lose plasma membrane integrity is not necessarily true for all kinds of necrotic cells at all developmental stages, and further suggest that for PS to be present on the surface of necrotic touch neurons, an active PS exposure mechanism must exist. Further genetic studies reported here revealed that at least two separate PS-exposure activities act to promote PS exposure on the surface of necrotic cells.

### PS as a common “eat me” signal elicited by necrotic and apoptotic cells and a ligand for phagocytic receptor(s)

PS is an evolutionarily conserved “eat me” signal exposed by apoptotic cells in metazoan organisms ranging from simple to complex and it is implicated in recruiting engulfing cells [27,29,43,63–65]. Here we report the active exposure of PS on the surface of necrotic touch neurons. The fact that both necrotic and apoptotic cells expose PS on their surfaces implies the existence of a conserved dying cell-recognition mechanism. Indeed, we further discovered the novel function of phagocytic receptor CED-1 in recognizing necrotic cells in addition to apoptotic cells. The extracellular domain of CED-1 (CED-1Ex) alone, without the transmembrane or intracellular domains, is sufficient for associating to the surface of necrotic cells, suggesting an extracellular ligand-receptor interaction. In support of this hypothesis, we have detected direct and selective *in vitro* interaction between CED-1Ex and acidic phospholipids including PS. On the other hand, CED-1Ex does not display affinity to phosphatidylcholine (PC), a neutral phospholipid without a net charge and is most abundant on the outer surface of the plasma membrane [32]. Consistent with this conclusion, Draper, the *Drosophila* orthologue of CED-1, also directly associates with PS exposed to the surface of apoptotic cells [29]. Together, the *in vivo* and *in vitro* observations indicate that the direct interaction between the CED-1 family phagocytic receptors and PS is an important mechanism that brings together phagocytes and their target cells, regardless of whether these cells die of apoptosis or necrosis.

As an “eat me” signal, PS is also known to attract phagocytic receptors via an indirect mechanism. Secreted bridging molecules such as mouse MFG-E8 bring dying and engulfing cells together by interacting simultaneously with both PS and phagocytic receptors [66]. *C*. *elegans* TTR-52, a transthyretin-like secreted protein, has been implicated as a bridging molecule that links PS on apoptotic cells to CED-1 on engulfing cell surfaces [67]. Our finding, together with that reported by Wang et al (2010) [67], indicate that direct and indirect interactions between PS and CED-1 provide two distinct molecular mechanisms to support the recognition of dying cells by CED-1.

The phosphatidylserine receptor (PSR) protein family was originally identified as PS receptors that promote apoptotic cell-removal yet was later reported to function in other developmental processes and possess several biochemical activities that are in conflict with the proposed role as PS receptors [68–71]. Recently, it was reported that *C*. *elegans* PSR-1 displayed an *in vitro* PS-binding affinity [72]. *In vivo*, *psr-1* mutants display weak defects in apoptotic- and necrotic-cell removal [72,73]. PSR-1 might thus contribute to the recognition of dying cells in addition to CED-1.

### CED-7 represents a common PS-exposure mechanism utilized by both apoptotic and necrotic cells

In *C*. *elegans*, CED-7, a member of the ABC transporter family, was known to regulate PS exposure by apoptotic cells [43]. Mouse ABCA1 was also reported to participate in PS redistribution during apoptotic cell clearance [40,74]. Our discovery of CED-7 as a key factor in promoting the externalization of PS by necrotic touch neurons further demonstrates that the presentation of the “eat me” signal shares conserved mechanism(s) during different types of cell death. We further discovered that CED-7 has two distinct functions, one in necrotic and the other in engulfing cells. How CED-7 acts in necrotic cells to promote PS exposure remains to be elucidated. CED-7 is ubiquitously expressed. There thus must be dying cell-specific mechanisms that activate CED-7. Whether the CED-7 activation mechanisms are common or distinct in necrotic and apoptotic cells remains unknown. Moreover, the engulfing cell-specific function of CED-7 is a mystery and requires further investigation. Previous research suggests that engulfing cells might also externalize PS and that ABC transporters might be involved in this event [40,75]. The function of this event remains to be clarified.

### A novel neuronal-specific PS-exposure mechanism represented by a Ca^2+^-dependent PS scramblase

Our observations indicate that ANOH-1, the *C*. *elegans* homolog of mammalian TMEM16F, functions in necrotic neurons to promote PS exposure. ANOH-1 is primarily expressed in neurons, including touch neurons [57] (Figs 5I and S5C). These lines of evidence indicate a cell type-specific function of ANOH-1 to facilitate necrotic-cell removal.

The vertebrates TMEM16 family of proteins, also known as anoctamins, are divided into two subfamilies based on two distinct Ca^2+^-dependent biochemical activities: Cl^-^ channels and lipid scramblases [37,76]. In addition, TMEM16F possesses both biochemical activities [36,77]. Mammalian TMEM16F promotes cellular PS exposure in response to Ca^2+^ ionophore yet not to apoptotic stimuli [37]. The Ca^2+^-activated phospholipid scramblase activity of the TMEM16 subfamily provides an important clue towards revealing a necrosis-specific PS-exposure mechanism (Fig 7C). As an evolutionarily conserved feature, Ca^2+^ influx is known to be an effective trigger of the excitotoxic death of mammalian neurons [78]. For example, the activation of the NMDA receptor upon binding to excessive glutamate elicits an initial rise of cytoplasmic calcium that induces a subsequent calcium-dependent calcium release from the ER [12,79,80]. Elevation of cytoplasmic Ca^2+^ is also a critical trigger for excitotoxic necrosis of neurons in *C*. *elegans*, including that of touch neurons and other types of neurons [24,81]. Particularly in touch neurons, the dominant mutation in MEC-4, a subunit of a multimeric, mechanically gated DEG/ENaC channel, leads to an increased influx of Ca^2+^, resulting in necrosis [19,22] (Fig 7C). We propose that in touch neurons that undergo Ca^2+^-activated necrosis, Ca^2+^ further acts as an activating factor for the PS-exposure activity of ANOH-1 (Fig 7C). This Ca^2+^-dependent PS-exposure mechanism might apply to multiple kinds of necrotic neurons including but not limited to mechanosensory neurons. Moreover, the possibility remains that CED-7 or CED-8 might also be activated by Ca^2+^ in necrotic neurons (Fig 7C). As the disruption of Ca^2+^ homeostasis is closely associated with neuron degeneration conditions [82], the work reported here has a broader application in understanding the physiological role of the clearance of many kinds of degenerative neurons resulted from pathological conditions or aging.

### Multiple activities contribute to PS exposure on the surface of necrotic cells

Our finding that the *anoh-1 ced-*7 double mutants display more severe defects in PS-exposure and necrotic cell-removal than each single mutant alone suggests that ANOH-1 and CED-7 together provide the necessary activities for efficient PS-exposure on necrotic touch neurons. One possible model is that they act in two independent and partially redundant pathways. The common function of CED-7 in both necrotic and apoptotic cells and the necrotic cell-specific function of ANOH-1 in facilitating PS-exposure have established that cells die of different mechanisms employ both common and unique molecular activities to present a common “eat me” signal. Given that a necrotic *C*. *elegans* neuron possesses a surface area many times of that of an apoptotic cell, the cooperation of multiple molecular activities such as those represented by CED-7 and ANOH-1, are likely essential for the efficient and timely exposure of PS on the cell surface at a level high enough to attract engulfing cells (Fig 7C). We further found that CED-8, a homolog of the mammalian phospholipid scramblase Xk8 [38,39], also made a modest contribution to the removal of necrotic cells. *ced-8* and *anoh-1* act in two independent pathways to promote PS exposure. Currently, it is unknown whether CED-8 facilitates PS-exposure to the surface of necrotic cells or whether CED-8 acts in necrotic cells; moreover, the functional relationship between *ced-7* and *ced-8* is unknown. CED-8 might represent a third pathway that is in parallel to both the CED-7 and the ANOH-1 pathways (Fig 7C).

## Materials and Methods

### Mutations, strains, and transgenic arrays

*C*. *elegans* was grown at 20°C as previously described [83] unless indicated otherwise. The N2 Bristol strain was used as the wild-type strain. Mutations are described in [84] and by the Wormbase (http://www.wormbase.org) unless noted otherwise: LGI, *ced-1(e1735)*, *ced-12(n3261)*; LGII, *enIs46[*P_*mec-7*_ *ced-7* and *punc-119(+)]*; LGIII, *ced-7* (*n1996*), *ced-6 (n2095)*, *anoh-1(tm4762)*, *unc-119(ed3);* LGIV, *ced-5(n1812)*, *ced-10(n1993);* LGV, *unc-76(e911)*, *deg-3(u662)*; LG X, *ced-8(n1891)*, *mec-4(e1611dm)*. The *tm4762* allele was generated and provided by the National Bioresource Project of Japan and was outcrossed twice prior to analysis. The precise location of nucleotide deletion has been confirmed by allele-sequencing. Integrated transgenic arrays used are as follows: LGII, *ttTi5605[mos]* [85]; LGV, *enIs33[Pdyn-1 mfg-e8*::*gfp* and *punc-76(+)]* [43].

Extrachromosomal arrays were generated by microinjection [86] of plasmids with coinjection marker p*unc-76(+)* [87] into strains carrying the *unc-76(e911)* mutant. Transgenic animals were isolated as non-Unc animals.

We obtained a single-copy insertion of *P*_*mec-7*_ *ced-7* in chromosome II in the ttTi5605 locus using the MosSCI insertion method [85], in strain EG4322 (*ttTi5605; unc-119(ed3))* [86]. The transgenic array also carries the *C*. *briggsae unc-119(+)* genomic DNA that rescues the *unc-119(ed3)* phenotype. The single-copy insertion of the transgenic array into anticipated locus was confirmed by single-worm PCR analysis.

### Plasmid construction

The P_*mec-7*_ *mrfp* (pZL08) and P_*mec-7*_ *mCherry* constructs were generated by replacing GFP in *P*_*mec-7*_ *gfp* (pPD117.01, a gift from Andrew Fire) with *mrfp* [88] or mCherry [89]. P_*col-10*_ *ssGFP* is a secreted GFP reporter expressed by hypodermal cells under the control of P_*col-10*_, the promoter for *col-10*. It is generated by replacing the *myo-3* promoter (P_*myo-3*_) in the P_*myo-3*_ *ssGFP* reporter [90] with P_*col-10*_, a gift from V. Ambros [28]. P_*ced-1*_ *ced-7* was constructed by placing the 5.1kb CED-7 cDNA [54] behind the P_*ced-1*_ promoter [28]. P_*mec-7*_ *ced-7* and *P*_*mec-7*_ *ced-7*::*gfp* were constructed by replacing P_*ced-1*_ from P_*ced-1*_ *ced-7* with the Sph-1-ClaI fragment of P_*mec-7*_ from pPD117.01, respectively. The P_*mec-7*_ *ced-7*/*unc-119(+)* construct for single-copy MosSCI insertion was generated by cloning the P_*mec-7*_ *ced-7* fragment into the BssHII and SpeI sites of plasmid CFJ151 [85].

The *anoh-1a* cDNA was amplified from total RNA from mixed-stage *C*. *elegans* population through RT-PCR and cloned into pPD117.01 to generate P_*mec-7*_ *gfp*::*anoh-1a* and *P*_*mec-7*_ *anoh-1a*::*gfp*. The *anoh-1b* genomic-cDNA chimeric fragment was constructed by ligating exon 1 and intron 1 of *anoh-1b* genomic DNA with *anoh-1a* cDNA, and similarly cloned into pPD117.01 to generate P_*mec-7*_ *gfp*::*anoh-1b* and *P*_*mec-7*_ *anoh-1b*::*gfp*. P_*ced-1*_ *anoh-1a*::*gfp* and P_*ced-1*_ *anoh-1b*::*gfp* were constructed by replacing P_*mec-7*_, respectively, with P_*ced-1*_ from pZZ829 [91]. The 617bp 5’ UTR of *anoh-1b* together with the first 297bp of exon 1 of the *anoh-1b* isoform was PCR-amplified from N2 worm extracts and cloned into pPD95.69 (a gift from Andy Fire), a promoter-less vector carrying a *gfp* cDNA tagged with a SV40 nuclear localization signal (NLS::gfp), between SphI and XmaI sites to generate P_*anoh-1b*_ *NLS*::*gfp*, which allowed us to identify the cells in which *anoh-1b* was expressed.

### RNA preparation for RT-PCR

Total RNA was isolated from mixed-stage *C*. *elegans* population using Trizol extraction with column purification (Qiagen, Inc.). cDNA was synthesized using the iScript cDNA Synthesis Kit (BIO-RAD, Inc.).

### Assays detecting *in vitro* interaction between CED-1 and phospholipids

The cDNA encoding the extracellular region of CED-1 tagged with GST at its C-terminus (CED-1-GST) was expressed in insect Sf9 cells using Bac-to-Bac Baculovirus Expression System, a baculovirus-based vector system, (Life Technologies Japan, Tokyo, Japan), and the resulting protein was affinity-purified by glutathione-Sepharose chromatography (GE Healthcare Japan, Tokyo, Japan), essentially as described previously [92]. An ELISA-like solid-phase binding reaction was conducted virtually according to the published procedure [93]. In brief, varying amounts of CED-1-GST or GST, the latter serving as a negative control, were added to the wells of a 96-well culture container surface-coated with phospholipids and incubated for 1 h at room temperature. The wells were washed, successively incubated with anti-GST antibody (Millipore, Inc.) and anti-mouse IgG antibody conjugated with horseradish peroxidase, and then subjected to a colorimetric reaction with *o*-phenylenediamine followed by the measurement of OD_490_. An assay for surface plasmon resonance was done with Biacore 3000 (GE Healthcare Japan) using the HPA chip pre-bound by liposomes, as described previously [29]. Liposomes were prepared using PC alone (PC-only liposome) or a mixture of PC and PS at a molar ratio of 7:3 (PS-containing liposome) as described previously [94]. Phospholipids were purchased from Avanti Polar Lipids (Alabaster, USA).

### DIC microscopy

According to the previous reports, among the six touch neurons, ALML and ALMR are born at ~450 min post-1^st^ cleavage, PLML and PLMR are born at ~510 min post 1^st^-cleavage, whereas AVM and PVM are born at ~9 hrs after hatching, at the L1 larval stage [46,47]. To determine the efficiency of necrotic cell clearance during all four larval stages, we chose to score the presence of necrotic PLML and PLMR, two touch neurons in the tail. L1, L2, L3, and L4 larvae were staged as larvae collected within 1 hr, 15–16 hrs, 24–25 hrs, and 33–34 hrs after embryos hatching, respectively. The total number of necrotic touch neurons in the tail of 10 worms was scored, and the mean of three repeats was calculated. The number of apoptotic cells were scored in embryos of different stages, in the head of young L1 larvae hatched within 1 hr, and in the gonad of adult hermaphrodites 48 hrs after the mid-L4 stage as described [48].

### Fluorescence microscopy

Olympus IX70-Applied Precision DeltaVision microscope equipped with Photometris Coolsnap digital camera and Applied Precision Softworx 5.0 software was used to acquire serial Z-stacks of fluorescence images at 0.5 μm intervals and to deconvolve these images of embryos and larvae [48]. To quantify the MFG-E8::GFP signal intensity on the surface of necrotic cells (I_n_), following deconvolution of z-stack images using the Applied Precision Softworx software, the necrotic cell surface was outlined by two closed polygons and the signal intensity in the area of the bigger polygon was subtracted with that of the smaller polygon. To normalize the signal intensity, the same two polygons were placed in the area neighboring the necrotic cell and the background fluorescence intensity (I_b_) was measured using the formula similar to that applied to necrotic cell surface. The relative signal intensity (I_r_) of MFG-E8::GFP enriched on the necrotic cell surface is represented as I_n_/I_b_. For each data point, at least 40 necrotic cells were quantified.

To monitor the dynamics of PS presentation during the necrosis of touch neurons in embryos via time-lapse recording, embryos were mounted on an agar pad on a glass slide in M9 solution [48]. The starting point of recording was at 460 min-post the 1st cleavage (the 1^st^ embryonic cell division), when an embryo reached the 2-fold stage. Recording was performed in 5-min interval until the embryo hatched. At each time point, a Z-stack of images composed of 40 serial Z sections at 0.5 μm/section were captured. Since embryos continue to move inside the eggshell, PLML and PLMR were followed by monitoring both the touch neuron reporter P_*mec-7*_ GFP and the distinct swelling morphology of necrotic cells.

For propidium iodide staining, mixed-stage worms were washed off plate using Hanks’ balanced salt solution buffer (HBSS buffer; with calcium and magnesium, Fisher Scientific) containing 10 μM propidium iodide and incubated for 2 hrs [95]. Worms were subsequently washed three times using HBSS buffer and mounted on an agar pad on a glass slide in 30 mM sodium azide for microscopic observation. Olympus IX70-Applied Precision DeltaVision microscope was used to acquire serial Z-stacks at 0.5 μm interval. Excitation and emission wavelengths used are ~540 and ~590 nm, respectively.

## Supporting Information

S1 Fig Necrotic touch neurons are not leaky. (A) The localization of four reporters, P_*mec-7*_ GFP (a, e) and P_*mec-7*_ mRFP (b, f), which are touch neuron-specific, and P_*col-10*_ ssGFP (c, g) and P_*myo-3*_ ssGFP (d, h), which are specifically expressed in hypodermal and body wall muscle cells, respectively, were individually analyzed in regards to necrotic touch neurons (arrowheads) in the tail of L1 larvae in the *mec-4(e1611dm)* background. Dorsal is to the top. Scale bars are 5μm. (B) The secreted GFP molecules expressed in body wall muscles and hypodermal cells under the P_*myo-3*_ or P_*col-10*_ promoters, respectively, are secreted as expected and are internalized by coelomocytes. Shown here are DIC/GFP merged (a, b) and corresponding DIC images (c, d) of adult *mec-4(e1611dm)* animals expressing GFP tagged with the signal sequence (ss) and under the control of P_*myo-3*_ (a, c) or P_*col-10*_ (b, d). White arrowheads indicate coelomocytes in which the ssGFP signal is detected. Scale bars are 10μm. (C) (a-d) DIC (a, c) and the corresponding propidium iodide staining (b, d) images of the tail region in wild-type and *mec-4(e1611)* L1 larvae. Arrows indicate the intestinal track. Arrowheads label necrotic cells. (e) Quantitative analysis of the percentage of necrotic cells stained with propidium iodide.
(TIF)

S2 Fig PS is detected on the surfaces of necrotic neurons in *deg-3(u662)* mutants. (A) DIC (a) and corresponding epifluorescence (b) images of MFG-E8::GFP in a *deg-3(u662)* mutant L1 larva. White arrowheads mark the AVG neuron that undergoes necrosis. Dorsal is up. Scale bars are 5μm. (B) The percentage of necrotic neurons labeled with MFG-E8::GFP on their surfaces (n = 20 animals).
(TIF)

S3 Fig Surface plasmon resonance assays also detect the specific interaction between CED-1-GST and PS. The binding of CED-1-GST to PS and PC, which were attached to the HPA chip as liposomes, was examined in an assay of surface plasmon resonance using Biacore 3000. (A) shows a change of the response unit (RU) during injection of liposomes and other substances, and (B) shows the binding of CED-1-GST to the chip coated with PS and PC. The arrows indicate time points of the injection of (a) PS-liposome 0.5 mM, (b) PC-liposome 0.5 mM, (c) 50 mM NaOH, (d) phosphate-buffered saline containing 0.1 mg/ml bovine serum albumin, and (e) CED-1-GST in 6.3 n mole.
(TIF)

S4 Fig Sequence alignment between the two isoforms of ANOH-1 and mouse TMEM16F. Numbers indicate amino acid positions. Residues identical or similar in ANOH-1 and TMEM16F are shaded in black or gray, respectively. Dashes indicate gaps. The predicted transmembrane domains in ANOH-1 are underlined and labeled as TM1-8. The truncated ANOH-1b(*tm4762*) peptide is listed, with the premature stop codon in red.
(TIF)

S5 Fig Detection of *anoh-1b* transcript and expression pattern. (A) Gene structure of the *anoh-1b* isoform. P1 to 12 are primers used in RT-PCR (B). The red open box and triangle indicate the region deleted in the *anoh-1(tm4762)* allele. (B) The *anoh-1b* mRNA is detected in *C*. *elegans* extract by RT-PCR. The RT-PCR products corresponding to *anoh-1b* mRNA were obtained by two rounds of PCR reactions (primary and nest PCRs). The genotype *anoh-1*(-) is short for *anoh-1(tm4762)*. In (b), *act-1*(F/R) represent the forward and reverse primers corresponding to *act-1*, a positive control included here to demonstrate that the mRNA prep and subsequent cDNA prep for both the wild-type and *anoh-1(tm4762)* mutant strains were of good quality and that equal amount of template was used for every sample. (C) Shown here are epifluorescence and the corresponding DIC images of the head and tail regions of a wild-type L4 larva co-expressing P_*rab-3*_ dsRed, a reporter that specifically marks neurons, and P_*anoh-1b*_ NLS::GFP. White arrowheads in (d to q) indicate cells marked by both GFP and dsRed. Arrows in (g, h, i) label pharyngeal neurons. Arrows in (o and r) label a touch neuron. The particular z-sections of each set of images are labeled. Dorsal is up. Scale bars are 6μm.
(TIF)

S6 Fig PS exposure is increased in L2 stage *anoh-1* mutant larvae. (A) The MFG-E8::GFP signal intensity on the surface of necrotic touch neurons was measured in young L2 larvae (16 hrs post-hatching). Relative signal intensity was represented by the ratio between GFP signal intensity on the surface of necrotic cells in the tail and in a nearby region in the same tail. “n” indicates the number of necrotic cells (each represented by a grey circle) analyzed. Red lines indicate the median value of each group of samples. The blue line indicates the position of ratio value 1.0, which represents the lack of signal enrichment on necrotic cell surfaces. “***”, p<0.001, Student *t*-test. (B) Mean values of MFG-E8::GFP signal intensity on the surface of necrotic cells in *mec-4* and *anoh-1; mec-4* animals at L1 and L2 larval stages.
(TIF)

S7 Fig PS externalization defect of the *anoh-1* mutant could be rescued by *anoh-1b* transgene under the control of P_*mec-7*_. (A) PS presentation is normal in *anoh-1* animals expressing P_*mec-7*_ *anoh-1b*::*mCherry*. DIC (a-c) and corresponding epifluorescence (d-f) images of the tails of newly (within 1hr) hatched L1 larvae expressing P_*dyn-1*_ *mfg-e8*::*gfp* showing different GFP signal in different backgrounds on necrotic cell surfaces. White arrowheads label necrotic corpses. Dorsal is up. Scale bars are 10μm. (B) Relative signal intensity of MFG-E8::GFP was calculated as the ratio between GFP signal intensity on surfaces of necrotic cells in the tail and that in a nearby region inside the same worm. Signal intensity measurement was performed using L1 larvae aged within 1-hr of hatching. Each grey circle represents one necrotic cell analyzed. “n” indicates the number of necrotic cells analyzed for each genotype. Red lines represent the median value of each group of sample.
(TIF)

S8 Fig Inactivating *ced-6* results in necrotic cell removal defect but does not affect PS presentation on necrotic cell surfaces. Alleles used: *ced-6(n2094)*, *mec-4(e1611dm)*. (A) Worms of indicated genotypes were scored at four larval stages for the persistence of necrotic corpses in their tails. Sixty animals of each genotype were scored in six groups of 10 worms. Error bars indicate standard deviations of each data point. “***”, “**”, and “*” represent *p* values that are <0.001, 0.001<*p*<0.01, and 0.01<*p*<0.05, respectively. (B) PS is normally presented to necrotic cell surfaces in *ced-6* mutants. DIC (a and c) and corresponding epifluorescence (b and d) images of the tails of newly (within 1hr) hatched L1 larvae expressing P_*dyn-1*_ *mfg-e8*::*gfp*. White arrowheads label necrotic corpses. Dorsal is up. Scale bars are 10μm. (C) Relative signal intensity of MFG-E8::GFP was calculated as the ratio between GFP signal intensity on surfaces of necrotic cells in the tail and that in a nearby region inside the same worm. Signal intensity measurement was performed using L1 larvae aged within 1-hr of hatching. Each grey circle represents one necrotic cell analyzed. “n” indicates the number of necrotic cells analyzed for each genotype. Red lines represent the median value of each group of sample. The blue line indicates the position where the relative GFP signal intensity is 1.0, meaning no GFP enrichment on necrotic cell surfaces.
(TIF)

S1 Movie The dynamic processes of touch neuron necrosis and the exposure of PS on the surface of necrotic neurons monitored by time-lapse microscopy. Related to Fig 1E. Time-lapse recording was performed in a *mec-4 (e1611dm)* embryo, starting at ~460 min post-1^st^ cleavage. The changes of PLML and PLMR, two touch neurons in the tail, were followed using the following three reporters: (1) P_*mec-7*_ GFP, for the detecting specification of the touch neurons, (2) DIC microscopy, for monitoring the morphological changes throughout the necrosis process, and (3) MFG-E8::mCherry, for detecting PS on the surface of necrotic and apoptotic cells. At each time point, 40 slices of Z-sections were collected, with an interval of 0.5 μm. Arrowheads indicate both living and necrotic touch neurons. Arrows indicate apoptotic cells.
(MOV)
