# Correction: Necrotic Cells Actively Attract Phagocytes through the Collaborative Action of Two Distinct PS-Exposure Mechanisms

**DOI:** 10.1371/journal.pgen.1005418

**Published:** 2015-07-17

**Authors:** Zao Li, Victor Venegas, Yuji Nagaoka, Eri Morino, Prashant Raghavan, Anjon Audhya, Yoshinobu Nakanishi, Zheng Zhou

In the Results section, under the subheading “ANOH-1 acts in touch neurons to promote the removal of necrotic touch neurons”, the sentence “In addition, P*mec-7 anoh-1b*::*gfp* also leads to the recovery of PS exposure on necrotic neuron surfaces (S7 Fig)” is incorrect. The sentence should read: “In addition, P*mec-7 anoh-1b*::*mCherry* also leads to the recovery of PS exposure on necrotic neuron surfaces (S7 Fig).”
