# Supplementary figures and images for: Necrotic Cells Actively Attract Phagocytes through the Collaborative Action of Two Distinct PS-Exposure Mechanisms
Source: PLoS Genet. 2015 Jun 10;11(6):e1005285. doi: 10.1371/journal.pgen.1005285 (PMC4464654; doi:10.1371/journal.pgen.1005285)

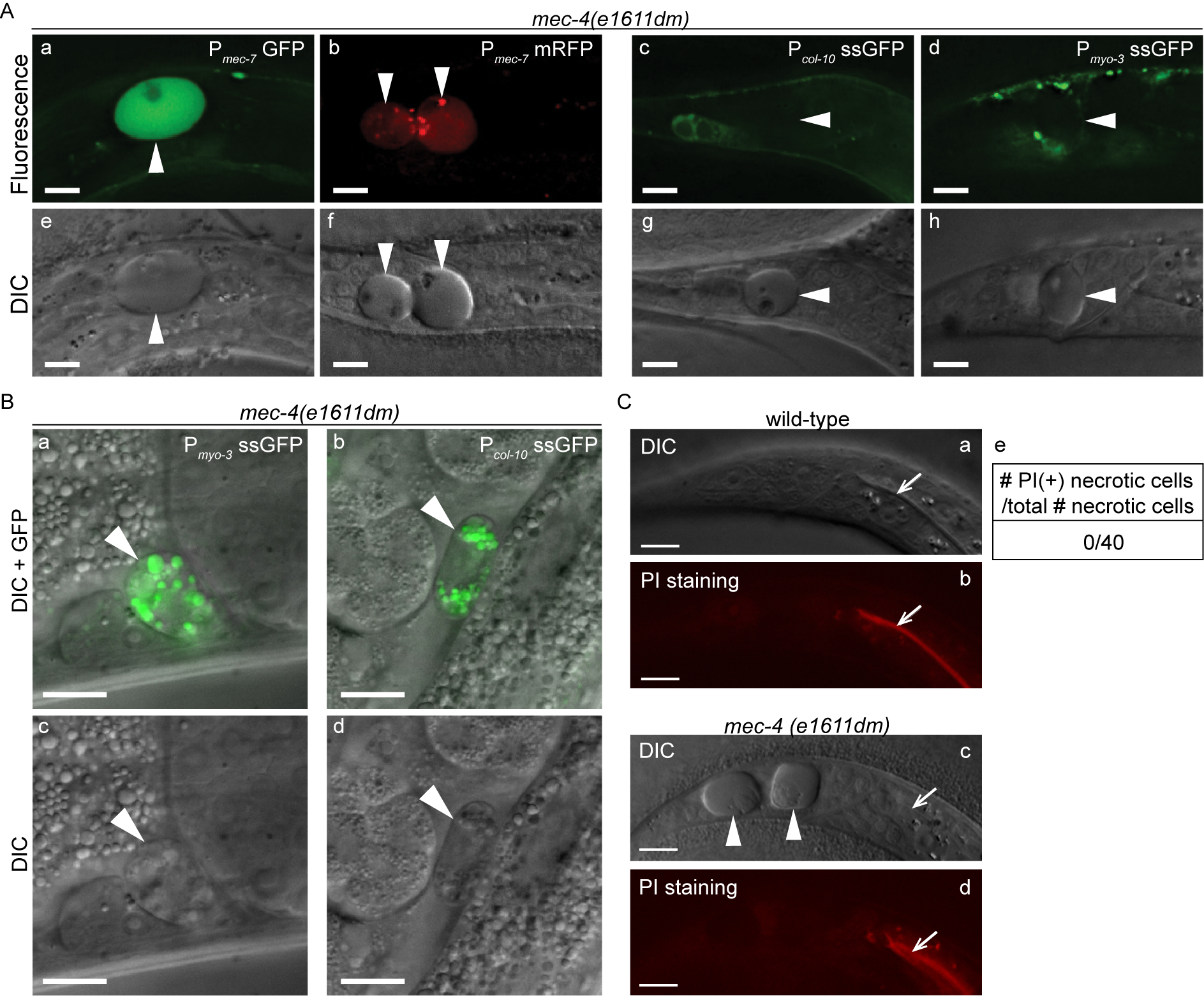

Supplement: S1 Fig — (A) The localization of four reporters, Pmec-7 GFP (a, e) and Pmec-7 mRFP (b, f), which are touch neuron-specific, and Pcol-10 ssGFP (c, g) and Pmyo-3 ssGFP (d, h), which are specifically expressed in hypodermal and body wall muscle cells, respectively, were individually analyzed in regards to necrotic touch neurons (arrowheads) in the tail of L1 larvae in the mec-4(e1611dm) background. Dorsal is to the top. Scale bars are 5μm. (B) The secreted GFP molecules expressed in body wall muscles and hypodermal cells under the Pmyo-3 or Pcol-10 promoters, respectively, are secreted as expected and are internalized by coelomocytes. Shown here are DIC/GFP merged (a, b) and corresponding DIC images (c, d) of adult mec-4(e1611dm) animals expressing GFP tagged with the signal sequence (ss) and under the control of Pmyo-3 (a, c) or Pcol-10 (b, d). White arrowheads indicate coelomocytes in which the ssGFP signal is detected. Scale bars are 10μm. (C) (a-d) DIC (a, c) and the corresponding propidium iodide staining (b, d) images of the tail region in wild-type and mec-4(e1611) L1 larvae. Arrows indicate the intestinal track. Arrowheads label necrotic cells. (e) Quantitative analysis of the percentage of necrotic cells stained with propidium iodide. (TIF) [file pgen.1005285.s001.tif]

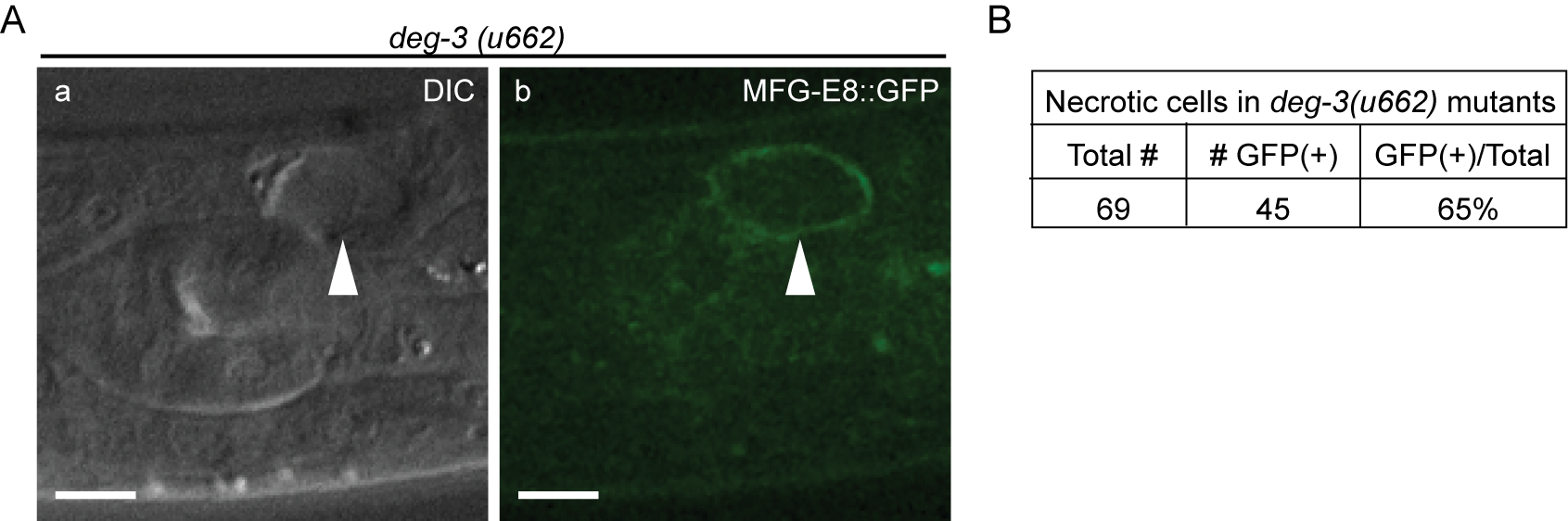

Supplement: S2 Fig — (A) DIC (a) and corresponding epifluorescence (b) images of MFG-E8::GFP in a deg-3(u662) mutant L1 larva. White arrowheads mark the AVG neuron that undergoes necrosis. Dorsal is up. Scale bars are 5μm. (B) The percentage of necrotic neurons labeled with MFG-E8::GFP on their surfaces (n = 20 animals). (TIF) [file pgen.1005285.s002.tif]

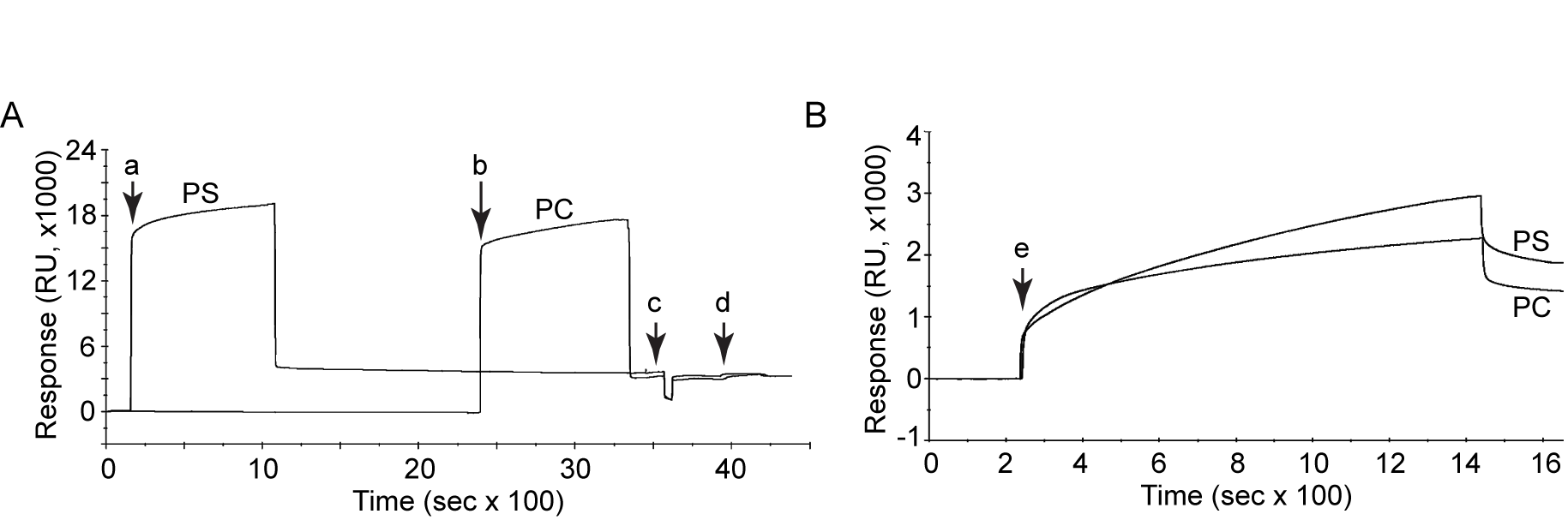

Supplement: S3 Fig — The binding of CED-1-GST to PS and PC, which were attached to the HPA chip as liposomes, was examined in an assay of surface plasmon resonance using Biacore 3000. (A) shows a change of the response unit (RU) during injection of liposomes and other substances, and (B) shows the binding of CED-1-GST to the chip coated with PS and PC. The arrows indicate time points of the injection of (a) PS-liposome 0.5 mM, (b) PC-liposome 0.5 mM, (c) 50 mM NaOH, (d) phosphate-buffered saline containing 0.1 mg/ml bovine serum albumin, and (e) CED-1-GST in 6.3 n mole. (TIF) [file pgen.1005285.s003.tif]

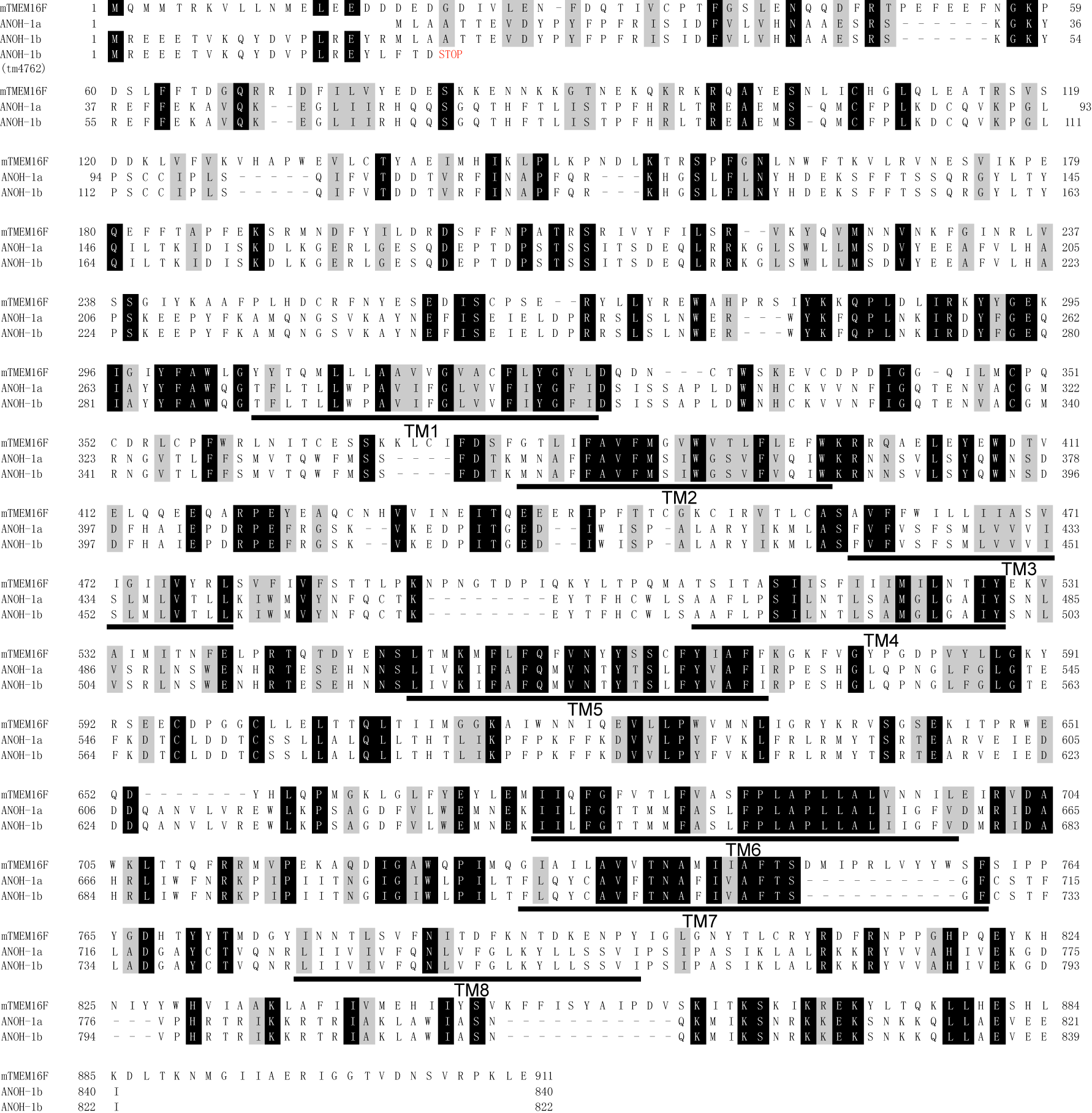

Supplement: S4 Fig — Numbers indicate amino acid positions. Residues identical or similar in ANOH-1 and TMEM16F are shaded in black or gray, respectively. Dashes indicate gaps. The predicted transmembrane domains in ANOH-1 are underlined and labeled as TM1-8. The truncated ANOH-1b(tm4762) peptide is listed, with the premature stop codon in red. (TIF) [file pgen.1005285.s004.tif]

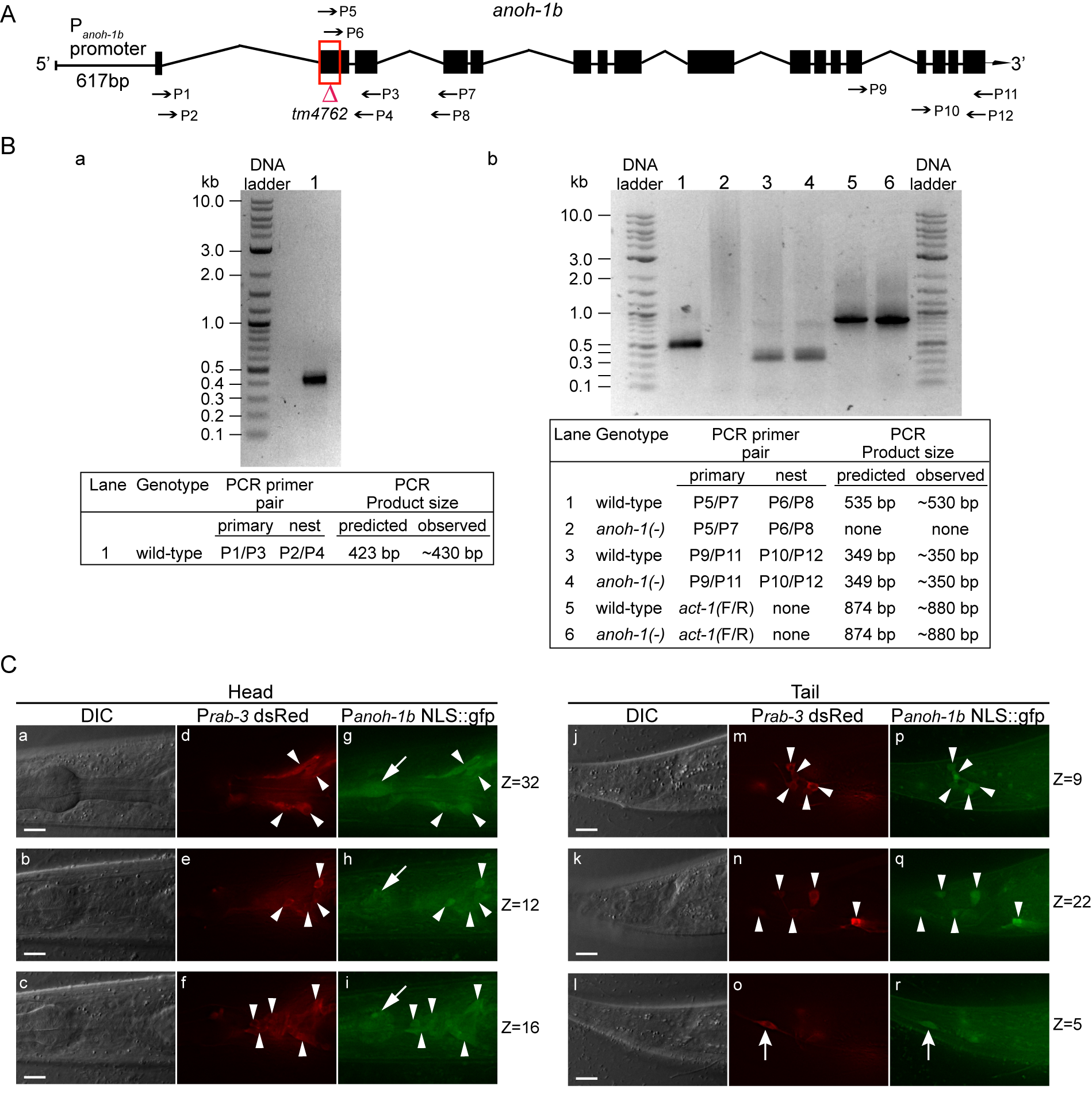

Supplement: S5 Fig — (A) Gene structure of the anoh-1b isoform. P1 to 12 are primers used in RT-PCR (B). The red open box and triangle indicate the region deleted in the anoh-1(tm4762) allele. (B) The anoh-1b mRNA is detected in C. elegans extract by RT-PCR. The RT-PCR products corresponding to anoh-1b mRNA were obtained by two rounds of PCR reactions (primary and nest PCRs). The genotype anoh-1(-) is short for anoh-1(tm4762). In (b), act-1(F/R) represent the forward and reverse primers corresponding to act-1, a positive control included here to demonstrate that the mRNA prep and subsequent cDNA prep for both the wild-type and anoh-1(tm4762) mutant strains were of good quality and that equal amount of template was used for every sample. (C) Shown here are epifluorescence and the corresponding DIC images of the head and tail regions of a wild-type L4 larva co-expressing Prab-3 dsRed, a reporter that specifically marks neurons, and Panoh-1b NLS::GFP. White arrowheads in (d to q) indicate cells marked by both GFP and dsRed. Arrows in (g, h, i) label pharyngeal neurons. Arrows in (o and r) label a touch neuron. The particular z-sections of each set of images are labeled. Dorsal is up. Scale bars are 6μm. (TIF) [file pgen.1005285.s005.tif]

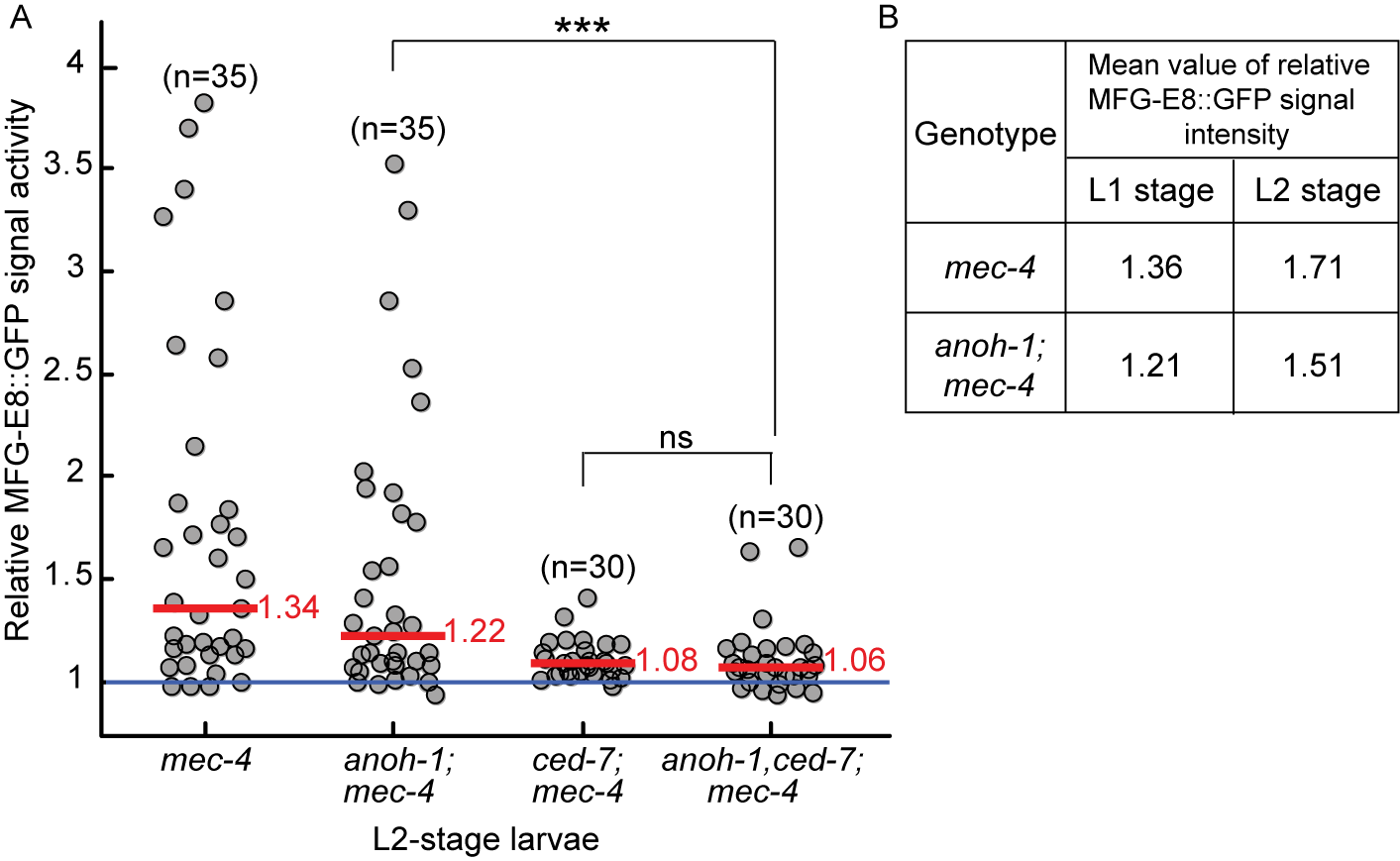

Supplement: S6 Fig — (A) The MFG-E8::GFP signal intensity on the surface of necrotic touch neurons was measured in young L2 larvae (16 hrs post-hatching). Relative signal intensity was represented by the ratio between GFP signal intensity on the surface of necrotic cells in the tail and in a nearby region in the same tail. “n” indicates the number of necrotic cells (each represented by a grey circle) analyzed. Red lines indicate the median value of each group of samples. The blue line indicates the position of ratio value 1.0, which represents the lack of signal enrichment on necrotic cell surfaces. “***”, p<0.001, Student t-test. (B) Mean values of MFG-E8::GFP signal intensity on the surface of necrotic cells in mec-4 and anoh-1; mec-4 animals at L1 and L2 larval stages. (TIF) [file pgen.1005285.s006.tif]

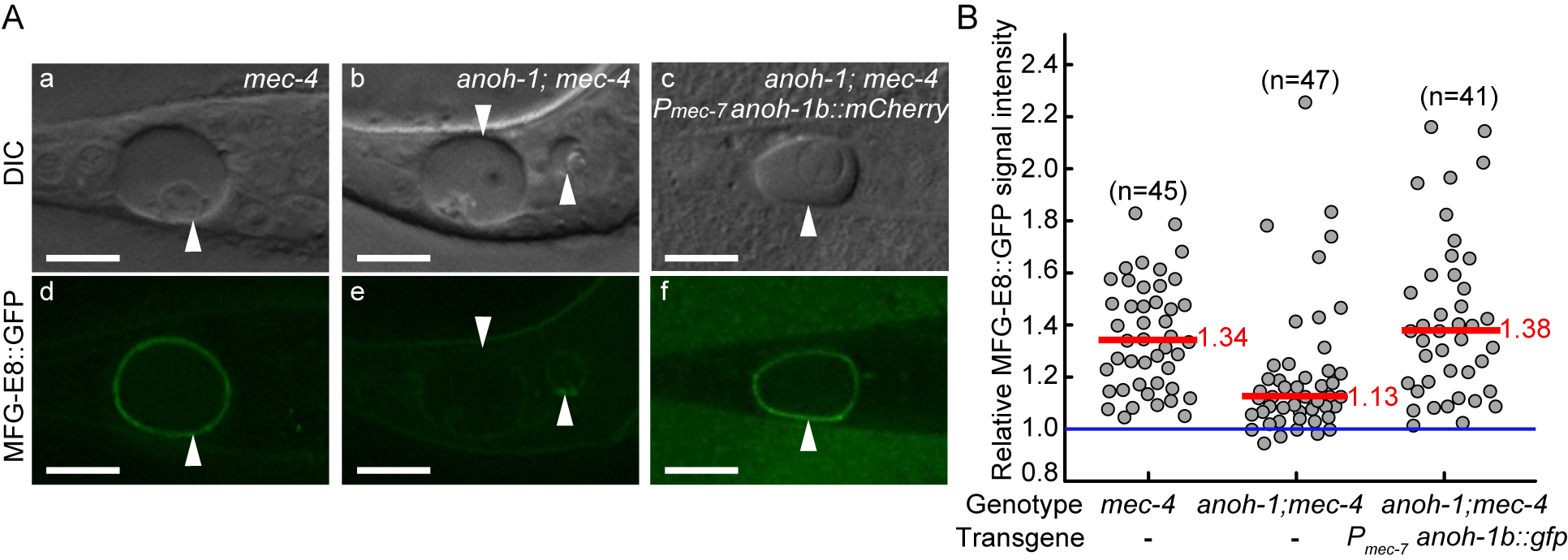

Supplement: S7 Fig — (A) PS presentation is normal in anoh-1 animals expressing Pmec-7 anoh-1b::mCherry. DIC (a-c) and corresponding epifluorescence (d-f) images of the tails of newly (within 1hr) hatched L1 larvae expressing Pdyn-1 mfg-e8::gfp showing different GFP signal in different backgrounds on necrotic cell surfaces. White arrowheads label necrotic corpses. Dorsal is up. Scale bars are 10μm. (B) Relative signal intensity of MFG-E8::GFP was calculated as the ratio between GFP signal intensity on surfaces of necrotic cells in the tail and that in a nearby region inside the same worm. Signal intensity measurement was performed using L1 larvae aged within 1-hr of hatching. Each grey circle represents one necrotic cell analyzed. “n” indicates the number of necrotic cells analyzed for each genotype. Red lines represent the median value of each group of sample. (TIF) [file pgen.1005285.s007.tif]

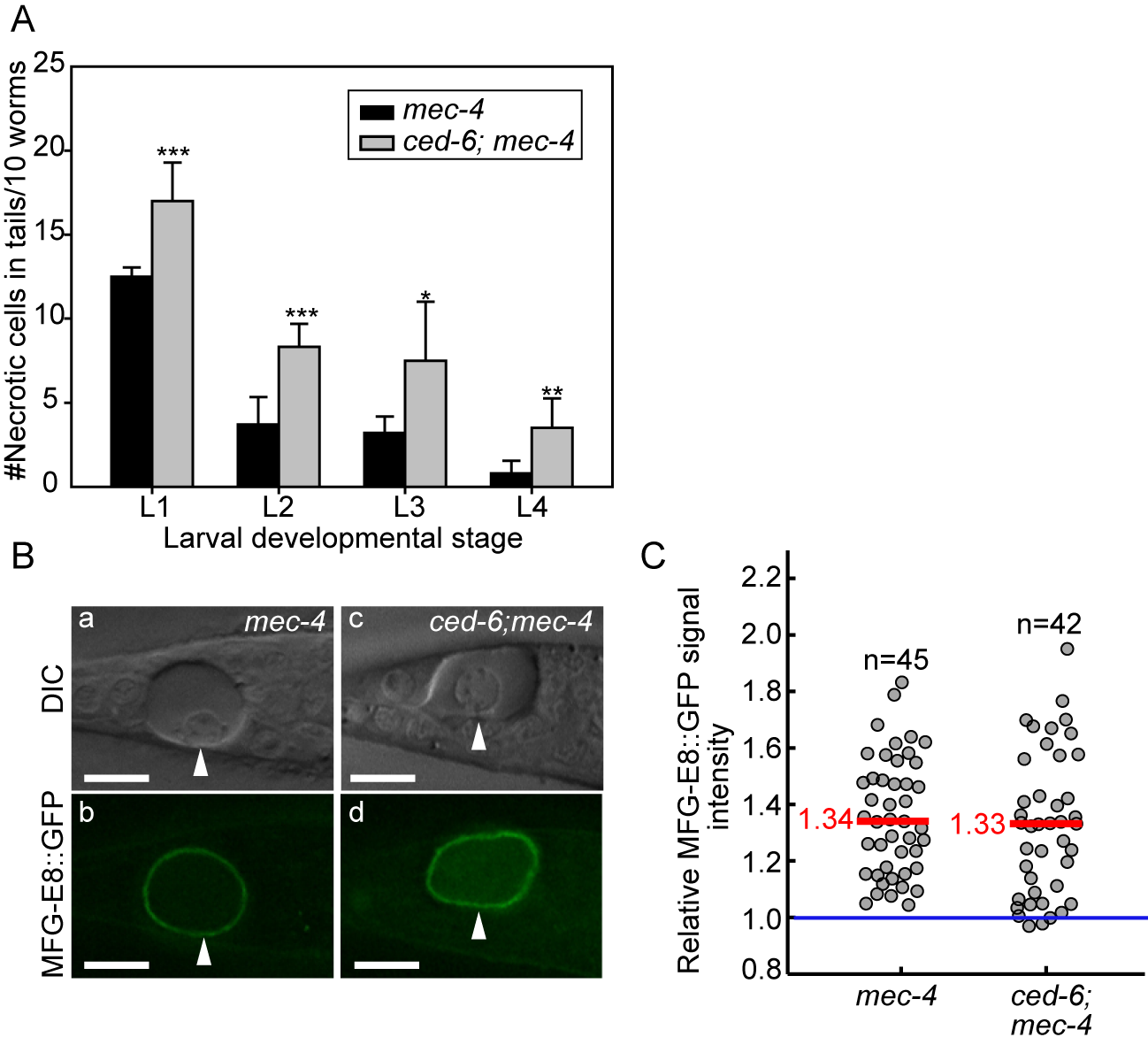

Supplement: S8 Fig — Alleles used: ced-6(n2094), mec-4(e1611dm). (A) Worms of indicated genotypes were scored at four larval stages for the persistence of necrotic corpses in their tails. Sixty animals of each genotype were scored in six groups of 10 worms. Error bars indicate standard deviations of each data point. “***”, “**”, and “*” represent p values that are <0.001, 0.001<p<0.01, and 0.01<p<0.05, respectively. (B) PS is normally presented to necrotic cell surfaces in ced-6 mutants. DIC (a and c) and corresponding epifluorescence (b and d) images of the tails of newly (within 1hr) hatched L1 larvae expressing Pdyn-1 mfg-e8::gfp. White arrowheads label necrotic corpses. Dorsal is up. Scale bars are 10μm. (C) Relative signal intensity of MFG-E8::GFP was calculated as the ratio between GFP signal intensity on surfaces of necrotic cells in the tail and that in a nearby region inside the same worm. Signal intensity measurement was performed using L1 larvae aged within 1-hr of hatching. Each grey circle represents one necrotic cell analyzed. “n” indicates the number of necrotic cells analyzed for each genotype. Red lines represent the median value of each group of sample. The blue line indicates the position where the relative GFP signal intensity is 1.0, meaning no GFP enrichment on necrotic cell surfaces. (TIF) [file pgen.1005285.s008.tif]
